# Orai1 calcium channel inhibition prevents progression of chronic pancreatitis

**DOI:** 10.1172/jci.insight.167645

**Published:** 2023-07-10

**Authors:** Viktória Szabó, Noémi Csákány-Papp, Marietta Görög, Tamara Madácsy, Árpád Varga, Aletta Kiss, Bálint Tél, Boldizsár Jójárt, Tim Crul, Krisztina Dudás, Mária Bagyánszki, Nikolett Bódi, Ferhan Ayaydin, Shyam Jee, László Tiszlavicz, Kenneth A. Stauderman, Sudarshan Hebbar, Petra Pallagi, József Maléth

**Affiliations:** 1 Department of Medicine,; 2 ELKH-USZ Momentum Epithelial Cell Signaling and Secretion Research Group, and; 3 HCEMM-USZ Molecular Gastroenterology Research Group, University of Szeged, Szeged, Hungary.; 4 First Department of Pediatrics, Semmelweis University, Budapest, Hungary.; 5 Department of Physiology, Anatomy and Neuroscience, Faculty of Science and Informatics,; 6 HCEMM-USZ Functional Cell Biology and Immunology Advanced Core Facility, and; 7 Department of Pathology, University of Szeged, Szeged, Hungary.; 8 CalciMedica Inc., La Jolla, California, USA.

**Keywords:** Cell Biology, Gastroenterology, Calcium channels, Calcium signaling, Fibrosis

## Abstract

Patients with recurrent acute pancreatitis (RAP) are at significant risk of developing early chronic pancreatitis (CP), which progresses into irreversible, end-stage CP with severe symptoms. There is no specific therapy in RAP or in early CP that may hinder disease progression. The pathogenesis of CP is complex and involves interactions among multiple cell types, including pancreatic acinar, ductal, and stellate cells (PSC). Therefore, it is pivotal to identify common pathogenic pathways in these cells that could be targeted pharmacologically. The Orai1-mediated store-operated Ca^2+^ entry (SOCE) is a ubiquitous signaling mechanism that may become overactivated in pathological states resulting in intracellular Ca^2+^ overload. In this study, we used ex vivo and in vivo preclinical disease models to demonstrate that Orai1 inhibition prevents progression of RAP and early CP. The selective Orai1 inhibitor CM5480 restored the expression of SOCE-associated regulatory factor in acinar cells, prevented uncontrolled Ca^2+^ elevation, protected acinar and ductal functions, mitigated immune cell infiltration, and diminished PSC activation, proliferation, and migration. We suggest that the overactivation of Orai1 is a crucial pathogenetic event in the progression of early CP and that inhibition of Orai1 could prevent the development of end-stage CP.

## Introduction

Chronic pancreatitis (CP) is a progressive inflammatory disease accompanied by functional and morphological damage of the pancreas (1, 2). Patients with CP suffer from malabsorption, postpancreatitis diabetes mellitus, debilitating pain, and a substantial decrease in the quality of life (2). In total, 20%–30% of patients with acute pancreatitis (AP) have a recurrence and ~10% develop CP (3), which is associated with an approximately 50% mortality rate within 20–25 years of diagnosis due to infection, malnutrition, and complications of recurrent acute pancreatitis (RAP) (4). Additionally, CP is the strongest identified risk factor for pancreatic cancer and increases the risk at least 13.3-fold (5), whereas patients with idiopathic and hereditary CP have a lifetime risk of ~50% to develop pancreatic ductal adenocarcinoma (6). In end-stage CP, the exocrine and endocrine pancreatic tissue is irreversibly damaged; thus, restoration of the pancreatic functions with pharmacological treatment is no longer possible. Consequently, the current clinical treatment is based on surgical and endoscopic interventions, pancreatic enzyme replacement, pain management, and nutrition (7), and there are no FDA-approved therapies for CP. The present disease progression model of CP suggests that a sentinel AP event is followed by RAP episodes leading to early CP, which progresses into established and end-stage disease (2). Whereas the pathological changes of the pancreas may be resolved in RAP and early CP, in established and end-stage CP, these are permanent. Therefore, an effective therapy applied in RAP or early CP may hinder the disease progression and prevent the development of an end-stage disease and address a significant unmet need.

The pathogenesis of CP is an intricated mechanism, in which genetic and environmental factors harm acinar and ductal cells and activate quiescent pancreatic stellate cells (PSCs) and immune cells. Given the complexity of CP, it is pivotal to identify common pathogenic events in these cell types that could be capitalized as potential drug targets. An obvious example for such mechanism is the intracellular Ca^2+^ signaling, which is a universal signal transduction pathway ubiquitously present in all eucaryotic cells — including pancreatic acinar and ductal cells and PSCs — as part of the physiological signaling process. Furthermore, sustained intracellular Ca^2+^ elevation is a hallmark of acute and chronic inflammatory diseases, such as different forms of pancreatitis (8). During the signaling process, agonist stimulation of G protein–coupled receptors or biological agents and toxins (like bile acids or fatty acid ethyl esters) release Ca^2+^ from the endoplasmic reticulum (ER) stores, and this is followed by the activation of stromal interaction molecule 1 (STIM1), an ER membrane Ca^2+^ sensor that triggers the influx of extracellular Ca^2+^ through plasma membrane Ca^2+^ channels including Orai proteins and canonical transient receptor potential channels (TRPC) (9). This process is termed store-operated Ca^2+^ entry (SOCE). To avoid uncontrolled Ca^2+^ influx, Orai1 is inactivated by another ER protein, the SOCE-associated regulatory factor (SARAF) (10). SARAF promotes fast and slow inactivation process of STIM2-dependent basal SOCE activity as well as STIM1-mediated SOCE activity in response to cytosolic Ca^2+^ elevation following ER Ca^2+^ refilling (10–12). Sarcoplasmic/endoplasmic and plasma membrane Ca^2+^ ATPase pumps are both activated by the rise in intracellular Ca^2+^ and fill the ER with Ca^2+^ and release cytoplasmic Ca^2+^, respectively. The periodic repetition of this cycle causes the physiological Ca^2+^ oscillations (13). Recently, Son et al. demonstrated that expression levels of SARAF decreased during AP (14), while, in other studies, selective inhibition of Orai1 markedly impaired bile acid or ethanol induced sustained Ca^2+^ overload in pancreatic acinar cells and decreased the severity of AP in mice (15, 16). Furthermore, our group lately showed that Orai1 inhibition is sufficient to maintain the ductal ion and fluid secretion during biliary or ethanol-induced AP in mice (17). These findings indicate that Orai1 has a pivotal role in acinar and ductal cell functions and emphasize it as a promising drug target, but the possible beneficial effects of Orai1 inhibition in RAP or early CP are currently unknown. Moreover, we have no information whether the selective disruption of the Orai1-mediated extracellular Ca^2+^ influx is sufficient to hinder the progression of acinar and ductal functional damage and reduce the extent of fibrosis in CP. Therefore, in this study, we aimed to comprehensively analyze the role of SOCE in the pathogenesis of RAP and early CP and assess the effect of Orai1 inhibition on the disease progression with a particular focus on the different cell types of the exocrine pancreas.

## Results

### SARAF expression is impaired in CP contributing to excess extracellular Ca^2+^ entry in pancreatic acinar cells.

**Figure 1:**
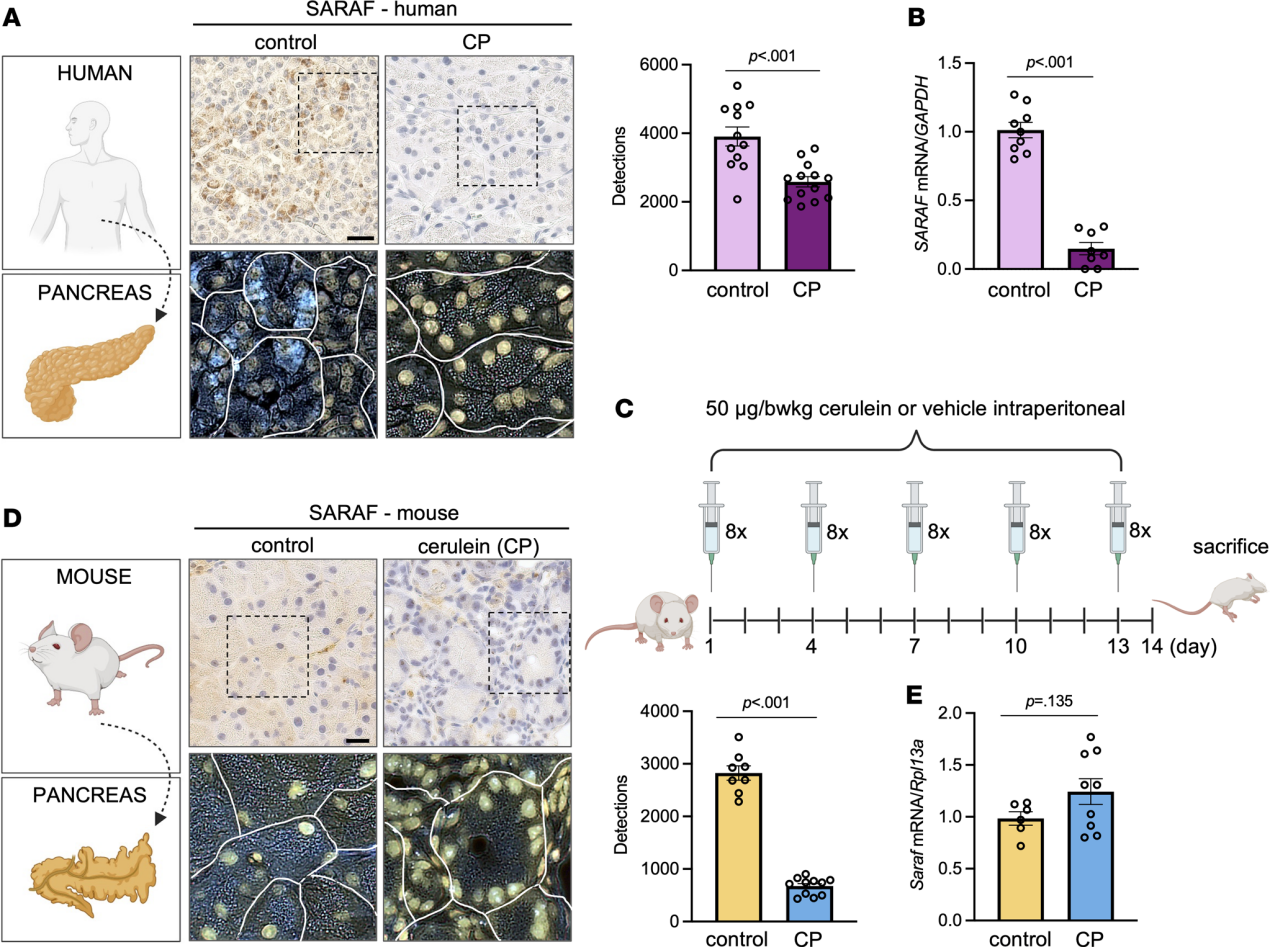
The amount of SARAF is decreased in CP in human and mice. (**A**) Representative IHC and inverted images of SARAF in the human pancreas. Scale bar: 20 μm. Bar chart demonstrates the number of detected pixels in control and CP samples (*n* = 3/group, 5–7 images/human). (**B**) *SARAF* mRNA expression in control and CP human pancreatic tissue (*n* = 3/group). (**C**) Representative scheme of the experimental setup showing the induction of RAP in mice by repetitive cerulein injections to trigger the development of early and established CP (5 series of 8 hourly i.p. cerulein injections every third day, 50 μg/bwkg). (**D**) Representative IHC and deconvoluted images of SARAF in the pancreas of control and CP mice (5 ***×*** 8 cerulein injections; 50 μg/bwkg). Scale bar: 20 μm. Bar chart demonstrates the number of detected pixels in control and CP samples (*n* = 3/group, 5–7 images/animal). (**E**) *Saraf* mRNA expression in control and CP mouse pancreatic tissue (*n* = 3/group). A *P* value less than 0.05 was considered significant by unpaired *t* test (**A**, **B**, **D**, and **E**). Data represent mean ± SEM. Explanatory images were created with BioRender. bwkg, body weight kilogram; CP, chronic pancreatitis.

**Figure 2:**
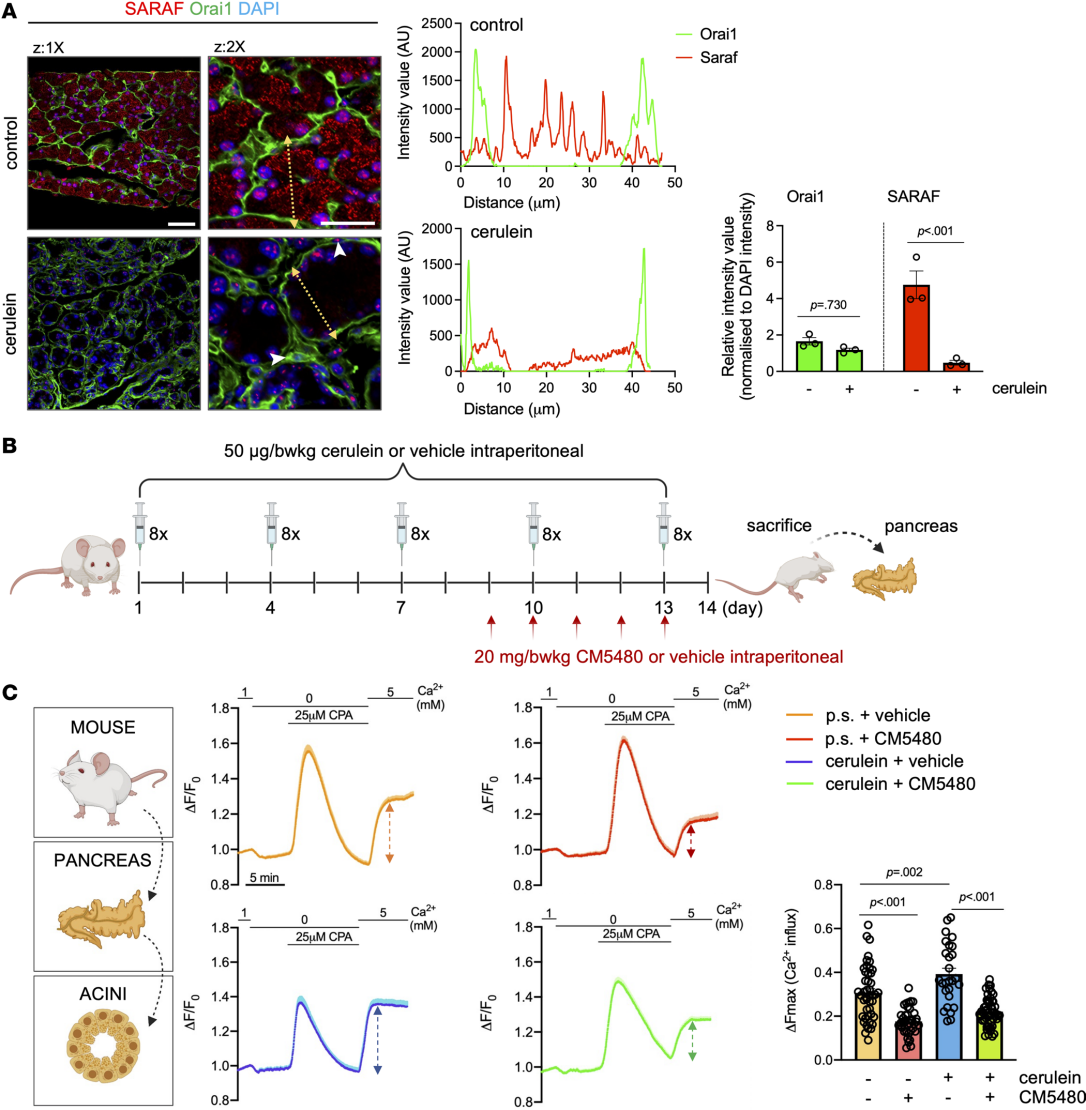
The decreased amount of SARAF is leading to increased extracellular Ca^2+^ entry. (**A**) Immunofluorescence staining and plot profiles of SARAF and Orai1 in control and cerulein-treated mouse pancreas. Scale bars: 50 μm. Yellow arrow: plot profile (*n* = 3/group, 5–7 images/animal). Bar charts indicate the quantified fluorescence intensity of Orai1 and SARAF normalized to DAPI. White arrowheads indicate the peri-acinar PSCs. (**B**) Representative scheme of the experimental setup of the induction of RAP by repetitive cerulein injections to trigger the development of early and established CP (5 series of 8 hourly i.p. cerulein injections every third day, 50 μg/bwkg) and CM5480 (20 mg/bwkg) treatment in mice. (**C**) Average traces of intracellular Ca^2+^ measurements in isolated pancreatic acinar clusters. The ER Ca^2+^ stores were depleted by CPA. The dashed arrows indicate the store-operated Ca^2+^ entry (maximal response) after readdition of the extracellular Ca^2+^. Bar chart summarizes the maximal response (*n* = 3/group; 5–7 individual experiments; data points in the bars represent individual cells). A *P* value less than 0.05 was considered significant by unpaired *t* test (**A**) and ordinary 1-way ANOVA (**C**). Data represent mean ± SEM. Explanatory images were created with BioRender. bwkg, body weight kilogram; CPA, cyclopiazonic acid; z, zoom.

The amount of SARAF is decreased in AP, whereas STIM1 and Orai1 remained unchanged (14), suggesting that SARAF might be more sensitive to pathological stimuli compared with other components of SOCE. In tissue samples from patients with CP, IHC revealed a remarkable decrease in the amount of SARAF in pancreatic acinar cells compared with the control pancreas (Figure 1A). Moreover, the mRNA expression of *SARAF* was decreased in the CP tissue samples as well (Figure 1B). For further investigations, we induced RAP in mice by repetitive cerulein injections to trigger the development of early and established CP (Figure 1C). In this model, the IHC showed that the amount of SARAF in pancreatic acinar cells was significantly lower in the cerulein-treated mice compared with the control (Figure 1D). Grayscale and magenta-colored merged image details also confirmed our findings (Supplemental Figure 1, A and B; supplemental material available online with this article; https://doi.org/10.1172/jci.insight.167645DS1). Whereas the mRNA expression of *Saraf* was not changed significantly upon cerulein treatment (Figure 1E), the subcellular distribution of SARAF in pancreatic acinar cells revealed a reticular intracellular expression pattern, presumably in the ER, while Orai1 localized mainly in the basolateral membrane of the control pancreatic acinar cells (Figure 2A and Supplemental Figure 1C). Moreover, the immunofluorescence staining confirmed that the amount of pancreatic SARAF remarkably decreased in the cerulein-treated mice, whereas the amount of Orai1 showed no detectable changes. Notably, Orai1 was present in the peri-acinar PSCs as well (white arrow heads). Control and cerulein-treated mice were treated with CM5480 during the last 5 days of CP induction; then, pancreatic acini were isolated (Figure 2B). To assess whether the decreased presence of SARAF has any impact on the intracellular Ca^2+^ homeostasis, we evaluated SOCE in isolated acinar clusters from control and cerulein-treated mice. SOCE was activated by the readdition of 5 mM extracellular Ca^2+^ after depletion of ER Ca^2+^ stores with 25 μM cyclopiazonic acid (CPA) in Ca^2+^-free solution (Figure 2C). Under these conditions, SOCE was significantly higher in the CP group. In contrast, when the mice were treated with 20 mg/body weight kilogram (bwkg) CM5480, SOCE was significantly reduced in the control and cerulein-treated animals as well. Based on these data, we conclude that the expression of SARAF significantly decreases in pancreatic acinar cells during CP, which could lead to excess extracellular Ca^2+^ influx by disturbed regulation of Orai1. Moreover, the increased Orai1-mediated Ca^2+^ entry can be prevented by Orai1 inhibition.

### Inhibition of Orai1 decreases the severity of CP in mice.

**Figure 3:**
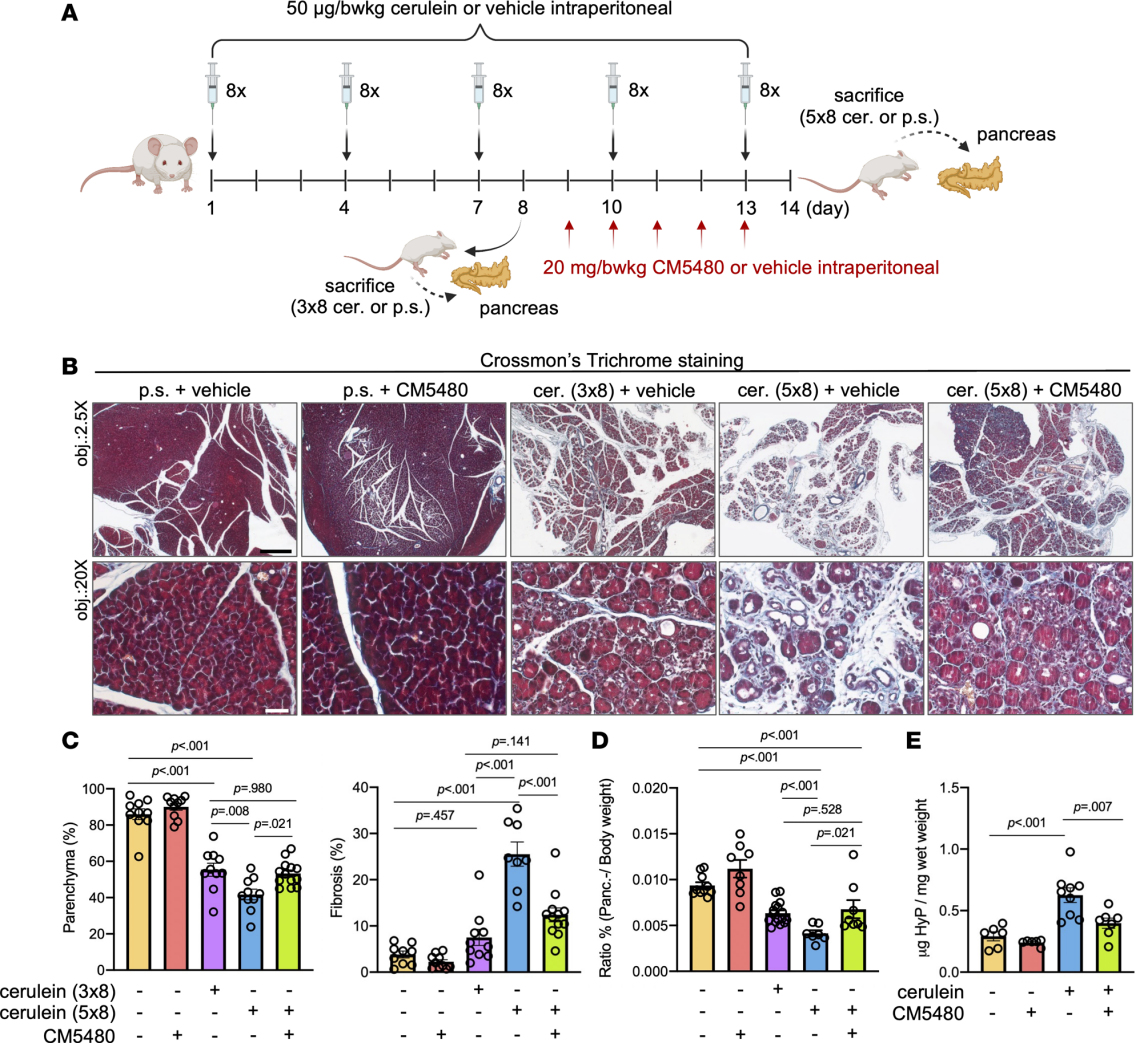
Orai1 inhibition reduces the severity of CP. (**A**) Representative scheme of the experimental setup of the induction of RAP by repetitive cerulein injections to trigger the development of early and established CP (3 or 5 series of 8 hourly i.p. cerulein injections every third day, 50 μg/bwkg) and CM5480 (20 mg/bwkg) treatment in mice. (**B**) Representative images of Crossmon’s trichrome staining of mouse pancreatic tissues. CP was induced by 3 ***×*** 8 and 5 ***×*** 8 cerulein (50 μg/bwkg), CM5480 was administrated on the last 5 consecutive days of the experiment. Scale bars: 500 μm (objective, 2.5***×***) or 50 μm (objective, 20***×***), respectively. (**C**) Bar charts of the tissue parenchyma and fibrosis (in ratio [%] of examined tissue type/whole tissue; *n* = 6–10/group, 5–7 images/animal). (**D**) Pancreas weight/body weight ratios. (**E**) HyP concentrations of the pancreas normalized to mg pancreas. For **C**–**E**, *n* = 6*–*10/group, 5–7 images/animal. A *P* value less than 0.05 was considered significant by ordinary 1-way ANOVA (**C** and **E**) and Mann-Whitney *U* test (**D**). Data represent mean ± SEM. Explanatory images were created with BioRender. bwkg, body weight kilogram; cer., cerulein; HyP, hydroxyproline; obj, objective; panc., pancreas; p.s., physiological saline.

**Figure 4:**
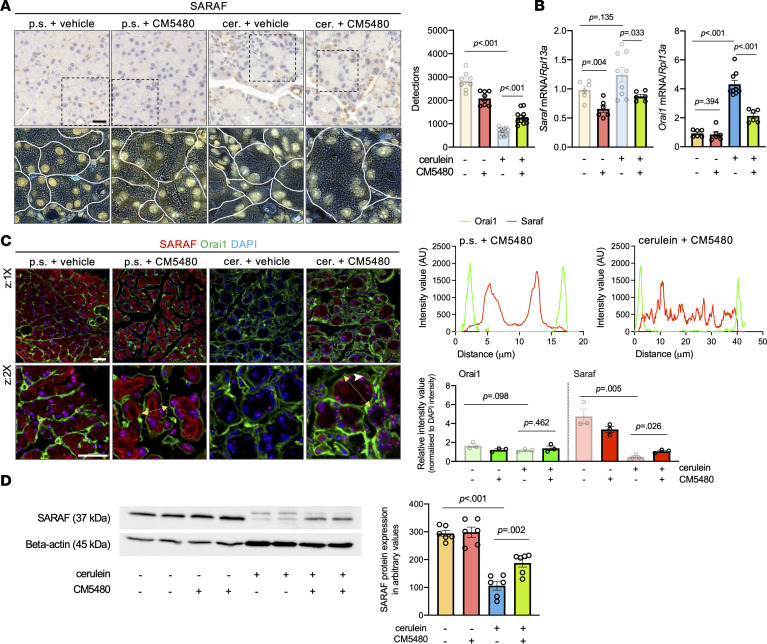
Localization and protein expression of SARAF partially restored upon Orai1 inhibition. (**A**) Representative IHC and deconvoluted images of SARAF in the pancreatic acini from control mice (p.s. + vehicle) and from mice treated with control + CM5480 (p.s. + CM5480), cerulein + vehicle, and cerulein + CM5480. Scale bar: 20 μm. Bar chart demonstrates the number of detections (*n* = 3/group, 5–7 images/animal). (**B**) SARAF and Orai1 mRNA expression in mouse pancreatic tissue (*n* = 3/group). (**C**) Immunofluorescence staining and plot profiles of SARAF and Orai1 in control (p.s. + vehicle) mice and in mouse pancreas treated with control + CM5480 (p.s. + CM5480), cerulein + vehicle and cerulein + CM5480 (*n* = 3/group, 5-7 images/animal). Scale bars: 50 m. Yellow arrow: plot profile. (**D**) Western blot analysis of SARAF and β-actin protein level: protein samples were isolated from mouse pancreas and loaded in 20 μg (*n* = 6/group). Bar charts represents the intensity analysis of SARAF protein expressions in arbitrary values. A *P* value less than 0.05 was considered significant by unpaired *t* test (**A** and **B** [*Saraf*], and **C**), Mann-Whitney *U* test (**B** [*Orai1*]), and ordinary 1-way ANOVA (**D**). Data represent mean ± SEM. cer., cerulein; p.s., physiological saline; z, zoom. Blurred bars on **A**–**C** are the data presented in Figure 1, D and E, and Figure 2A and are presented here for easier comparison. The statistical analysis in Figure 1, D and E; Figure 2A; and **A**–**C** account for all the comparisons made with the control data.

The augmentation of Orai1 function due to impaired SARAF protein expression may contribute to the cell damage in early CP; therefore, we hypothesized that the inhibition of Orai1 would decrease the severity of CP. Three episodes of RAP (8 cerulein injections on day 1, 4, and 7 before sacrifice [3 ***×*** 8 cerulein injections]) (Figure 3A) resulted in clear signs of the early CP, including acinar cell atrophy, fibrosis, and the presence of acinar-to-ductal metaplasia. Whereas after 5 ***×*** 8 cerulein injections (8 cerulein injections at day 1, 4, 7, 10, and 13 before sacrifice) the histology corresponded to established CP with severe atrophy and fibrosis (Supplemental Figure 2). This was further confirmed by Crossmon’s trichrome staining, which also revealed that 3 ***×*** 8 cerulein decreased the parenchyma percent and moderately increased tissue fibrosis (Figure 3, B and C). In addition, the pancreas weight/body weight ratio also reduced upon 3 ***×*** 8 cerulein treatment (Figure 3D). These parameters were significantly worsened in the mice treated with 5 ***×*** 8 cerulein injections accompanied by the weight loss of these animals (Supplemental Figure 3), suggesting the progression of the chronic inflammation. Importantly, in vivo administration of CM5480 significantly improved all parameters. No significant difference was detected between the mice receiving 3 ***×*** 8 cerulein injections (early CP) and the mice treated with 5 ***×*** 8 cerulein injections + CM5480, suggesting that the inhibition of Orai1 efficiently prevents the progression of early CP to established CP. The extent of fibrosis was also determined by the measurement of the pancreatic hydroxyproline (HyP) concentration, which is a major component of collagen. In line with the histology parameters, 5 ***×*** 8 cerulein injections significantly increased the pancreatic HyP concentration, which was lowered by CM5480 (Figure 3E). Next, we wanted to assess whether the inhibition of Orai1 restores the impaired SARAF protein expression observed in CP. Our results show that the in vivo treatment with CM5480 restored SARAF protein expression in pancreatic acinar cells of cerulein-treated mice (Figure 4A and Supplemental Figure 4A), whereas it had no relevant effect on the *Saraf* mRNA expression (Figure 4B). In contrast, gene expression of *Orai1* was significantly elevated in CP, which was decreased by the CM5480 treatment. Markedly, the elevated Orai1 gene expression was not translated to elevated protein expression (Figure 4B), suggesting that it may be compensated by other mechanisms. Notably, we observed an altered intracellular localization of SARAF, which translocated to the apical part of the acinar cells from the reticular localization pattern upon Orai1 inhibition (Figure 4C and Supplemental Figure 4B). This could be explained by the redistribution of ER to the apical pole of the acinar cells (18). More importantly, the amount of detectable SARAF was significantly increased in the cerulein-treated mice upon Orai1 inhibition These observations were also supported by Western blot analysis, which confirmed that the pancreatic expression of the SARAF is remarkably impaired by cerulein treatment, while CM5480 administration partially restored the protein expression. β-Actin was used as an internal control. It was previously described that the increased number of inflammatory cells result in the elevated expression of β-actin protein (19), which was also detected in this case (Figure 4D and Supplemental Figure 4C; see complete unedited blots in the supplement). Thus, inhibition of Orai1 with CM5480 restored the impaired expression of SARAF in pancreatic acinar cells and remarkably reduced the severity of CP by diminishing or even abolishing disease progression.

### Orai1 inhibition impairs the tissue inflammation in CP.

**Figure 5:**
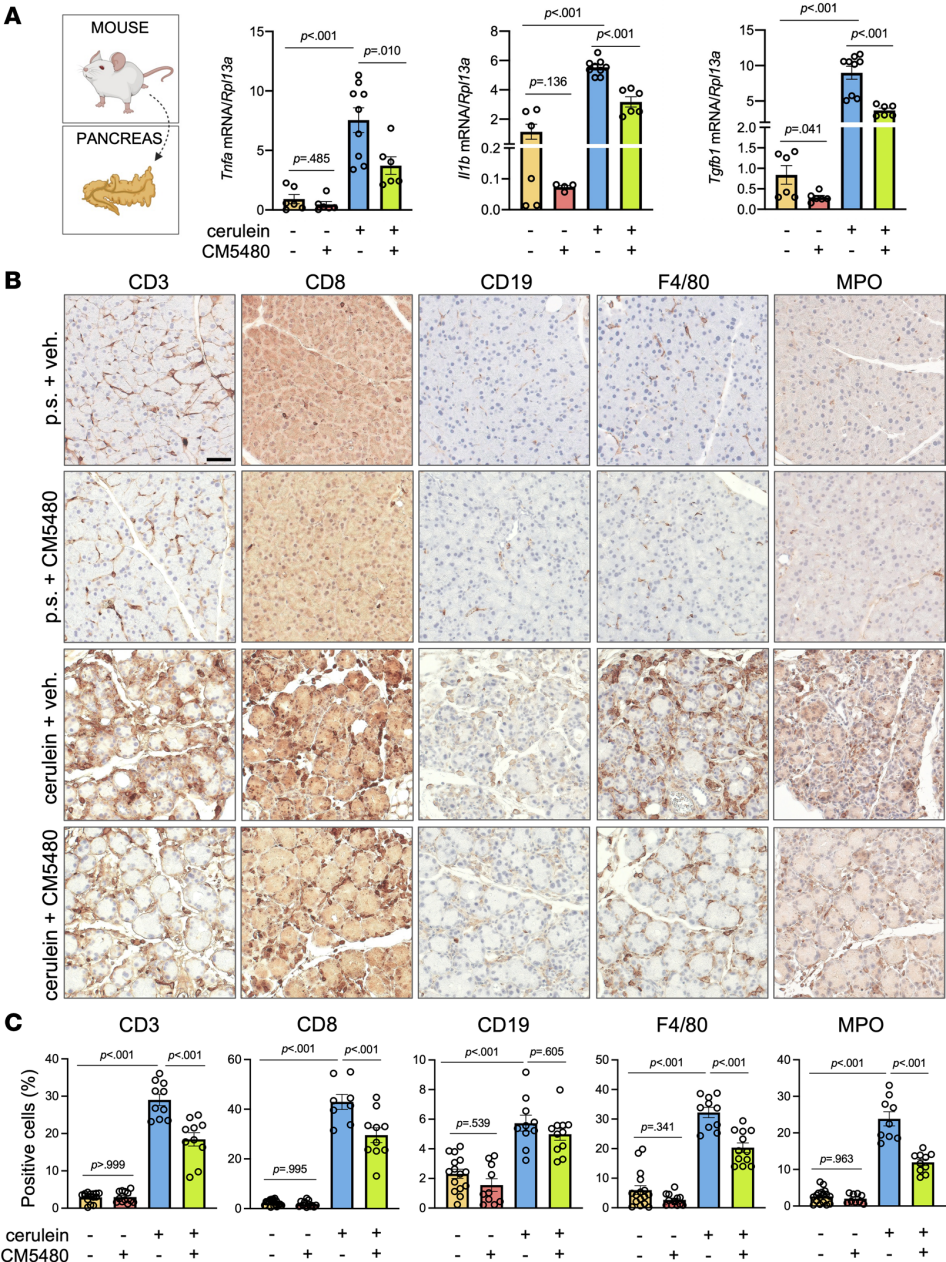
Orai1 inhibition decreases the inflammation in CP in mice. (**A**) *Tnfa*, *Il1b*, and *Tgfb1* mRNA expression in mouse pancreatic tissue (*n* = 3/group). (**B**) Representative IHC images of immune cell markers in mouse pancreas (CD3, cytotoxic and Th cell; CD8, cytotoxic T cell; CD19, B-lymphocyte marker; F4/80, macrophage; MPO (myeloperoxidase), neutrophil granulocyte). (**C**) Ratio of positive cells was determined by positive cells/total cells ratio (*n* = 3/group, 5–7 images/animal). Scale bar: 50 μm. A *P* value less than 0.05 was considered significant by unpaired *t* test (**A** [*Tnfa, Il1b*]) and Mann-Whitney *U* test (**B** [*Tgfb1*]) and ordinary 1-way ANOVA (**C**). Data represent mean ± SEM. Explanatory images were created with BioRender. p.s., physiological saline; veh., vehicle.

Chronic inflammation is the hallmark of CP that leads to permanent tissue damage and loss of pancreatic functions (20); therefore, we assessed the effect of Orai1 inhibition on the proinflammatory cytokine expression and immune cell infiltration in the pancreas. As expected, mRNA levels of proinflammatory cytokines — tumor necrosis factor α (*Tnfa*), IL-1b (*Il1b*), and TGF-β1 (*Tgfb1*) — were significantly elevated in cerulein-treated mice (Figure 5A) and were markedly lowered by the administration of CM5480 in the CP group. Notably, the inhibition of Orai1 decreased the expression of *Il1b* and *Tgfb1* in the control animals as well, and this could be explained by the role of Orai1 in the immune system (21). To decipher the immune cell infiltration during CP, the percentage of CD3^+^ cytotoxic and Th cells, CD8^+^ cytotoxic T-cells, CD19^+^ B-lymphocytes, F4/80^+^ macrophages, and myeloperoxidase-positive (MPO) neutrophil granulocytes were determined and normalized to the total cell numbers in a field of view (Figure 5, B and C). Similar to the mRNA expression results, we detected a remarkable elevation of immune cell infiltration of the pancreas in CP, which was significantly impaired by Orai1 inhibition, whereas CM5480 had no significant effect in the control animals. These results indicate that inhibition of Orai1 reduces proinflammatory immune cell infiltration and cytokine expression, thereby alleviating the inflammatory processes in CP.

### CM5480 significantly improves the diminished acinar and ductal cell functions in CP.

**Figure 6:**
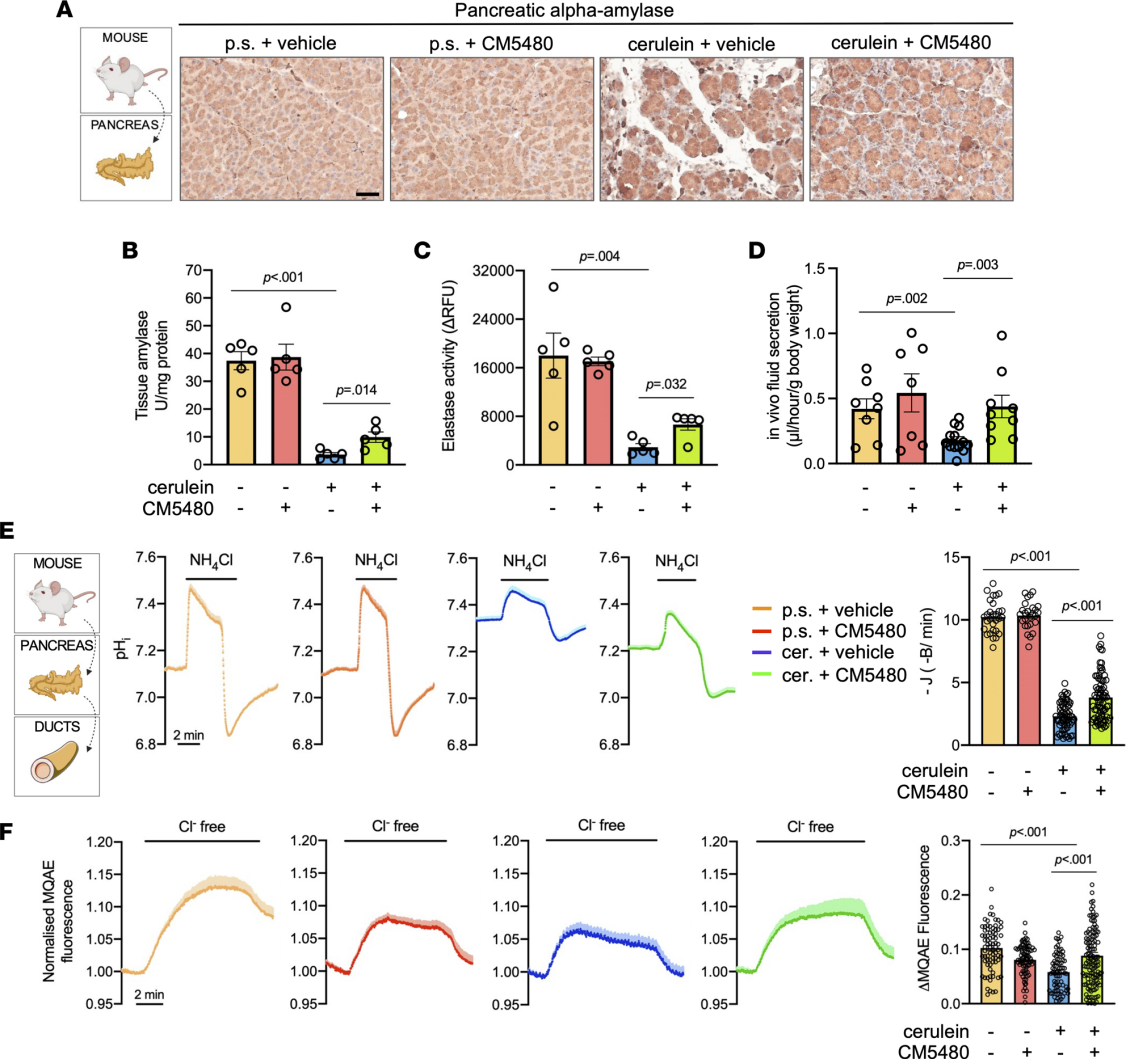
Orai1 inhibition ameliorates acinar and ductal functions. (**A**) Representative IHC images of pancreatic α-amylase in mouse pancreatic tissues (*n* = 3/group, 5-7 images/animal). Scale bar: 50 μm. (**B** and **C**) Tissue α-amylase and elastase activities normalized to the total protein amount (*n* = 5/group). (**D**) In vivo pancreatic fluid secretion (*n* = 7–10/group). (**E**) Average traces of the intracellular pH (pH_i_) of mouse pancreatic ducts perfused with 20 mM NH_4_Cl in HCO_3_^–^/CO_2_ buffered solution. The bar chart summarizes the base fluxes of HCO_3_^−^ calculated from the pH recovery after intracellular alkalization (*n* = 3/group and 5–7 individual experiments; data points in the bars indicate individual regions of interests). (**F**) Average traces of intracellular Cl^−^ levels. Removal of extracellular Cl^−^ reduced the intracellular Cl^−^ levels (reflected by an increase in fluorescence intensity) due to CFTR activity. The bar chart summarizes maximal intensity changes (ΔMQAE fluorescence; *n* = 3/group and 5–7 individual experiments; data points in the bars indicate individual regions of interests). A *P* value less than 0.05 was considered significant by unpaired *t* test (**B**–**D**) and Mann-Whitney *U* test (**E** and **F**). Data represent mean ± SEM. Explanatory images were created with BioRender. p.s., physiological saline.

**Figure 7:**
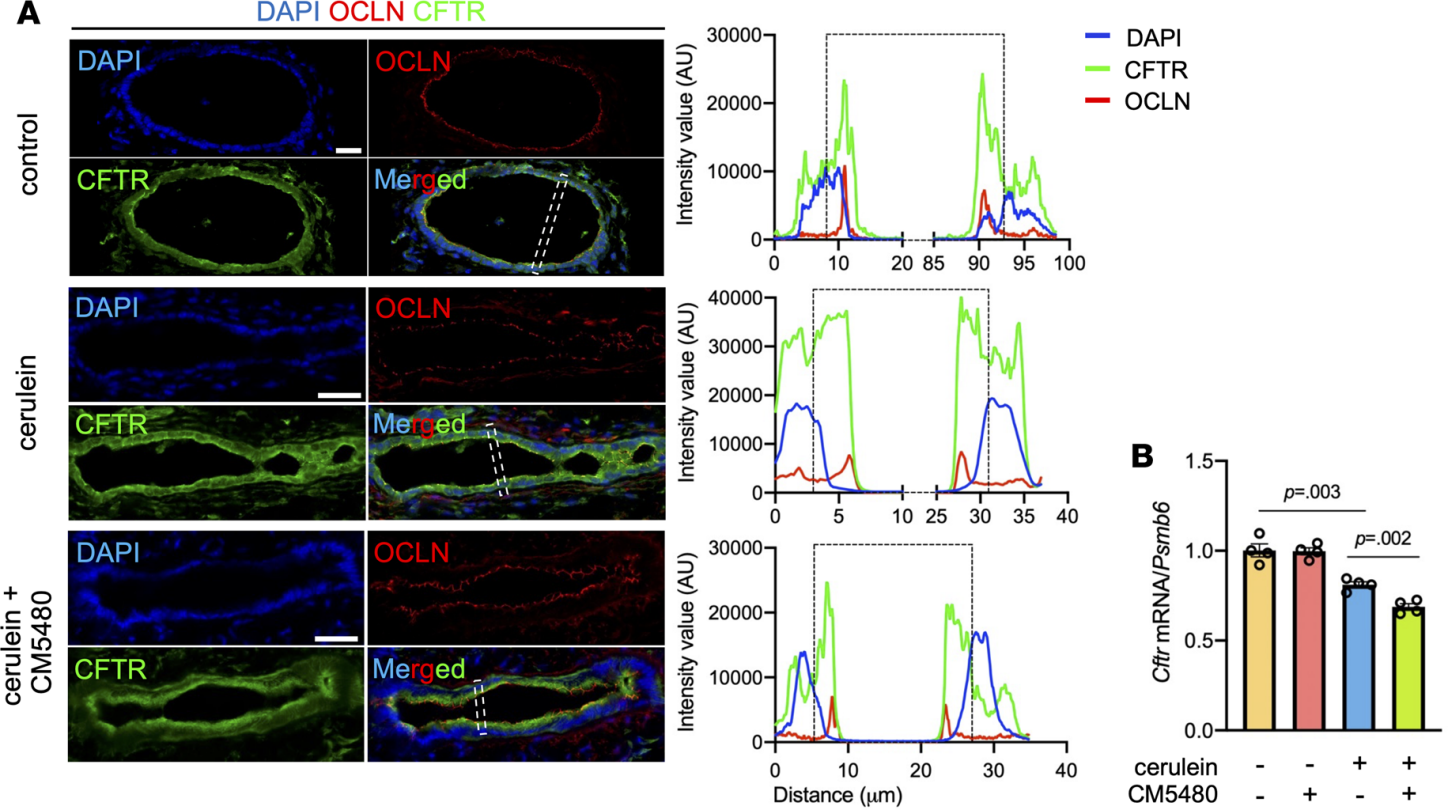
CM5480 treatment restores CFTR localization in CP. (**A**) Immunofluorescence staining and plot profile analysis of CFTR and Occludin (OCLN) in cross sections of isolated ductal fragments (*n* = 3/group, 5–7 images/animal). Scale bar: 20 μm. The areas delimited by dashed lines indicate the representative part from which the plot profile is derived. (**B**) *Cftr* mRNA expression in mouse pancreatic tissue (*n* = 3/group). A *P* value less than 0.05 was considered significant by unpaired *t* test (**B**). Data represent mean ± SEM.

Next, we wanted to clarify whether the impaired inflammation and tissue fibrosis is sufficient to maintain crucial exocrine pancreatic functions, such as acinar cell enzyme production, or ductal ion and fluid secretion. IHC and enzyme activity measurements revealed that the tissue α-amylase and elastase activities were significantly diminished in mice treated with cerulein, which was significantly improved by Orai1 inhibition in the CP animals but not affected in the controls (Figure 6, A–C, and Supplemental Figure 5). This indicates that the number of functional acini was highly decreased in CP but significantly improved by CM5480 treatment. Additionally, secretin-stimulated in vivo ductal fluid secretion was significantly impaired by cerulein treatment (Figure 6D). In contrast, this was completely restored to the control level in mice treated with cerulein and CM5480. To measure ductal HCO_3_^–^ secretion, pancreatic ductal fragments were isolated from mice in each experimental group and intracellular alkalization was triggered with the administration of 20 mM NH_4_Cl in HCO_3_^–^/CO_2_ buffered extracellular solution (22). Under these conditions, the inhibition of Orai1 had no effect on the HCO_3_^–^ extrusion in the control group, whereas cerulein treatment significantly diminished the ductal HCO_3_^–^ secretion, which was prevented by CM5480 treatment (Figure 6E). The mislocalization and impaired function of CFTR in pancreatic ductal cells are known characteristics of CP (23); therefore, we also investigated the effect of Orai1 inhibition on these features. The intracellular Cl^–^ levels were investigated with MQAE (N-(Ethoxycarbonylmethyl)-6-Methoxyquinolinium Bromide), which reflects the CFTR-mediated Cl^–^ extrusion (18). Removal of extracellular Cl^–^ increased the fluorescence intensity, which was significantly reduced by cerulein (Figure 6F). In vivo CM5480 treatment improved the cerulein-induced decrease significantly, suggesting a restoration of CFTR function. Immunolocalization revealed that, in control ducts, CFTR and Occludin (used as an apical membrane and barrier integrity marker) localized to the apical surface; however, in the cerulein-treated mice, a translocation of CFTR was observed into the cytoplasm of ductal epithelial cells (Figure 7A). Importantly, inhibition of Orai1 was able to restore the apical plasma membrane localization of CFTR. Finally, analysis of *Cftr* mRNA expression showed a statistically measurable decrease in the cerulein-treated and cerulein + CM5480–treated groups, but these are unlikely biologically meaningful alterations (Figure 7B). In summary, our data demonstrate that the inhibition of Orai1 can prevent the damage of pancreatic acinar and ductal cells and restores the physiologically essential exocrine pancreatic functions.

### CM5480 inhibits the activation of PSCs.

**Figure 8:**
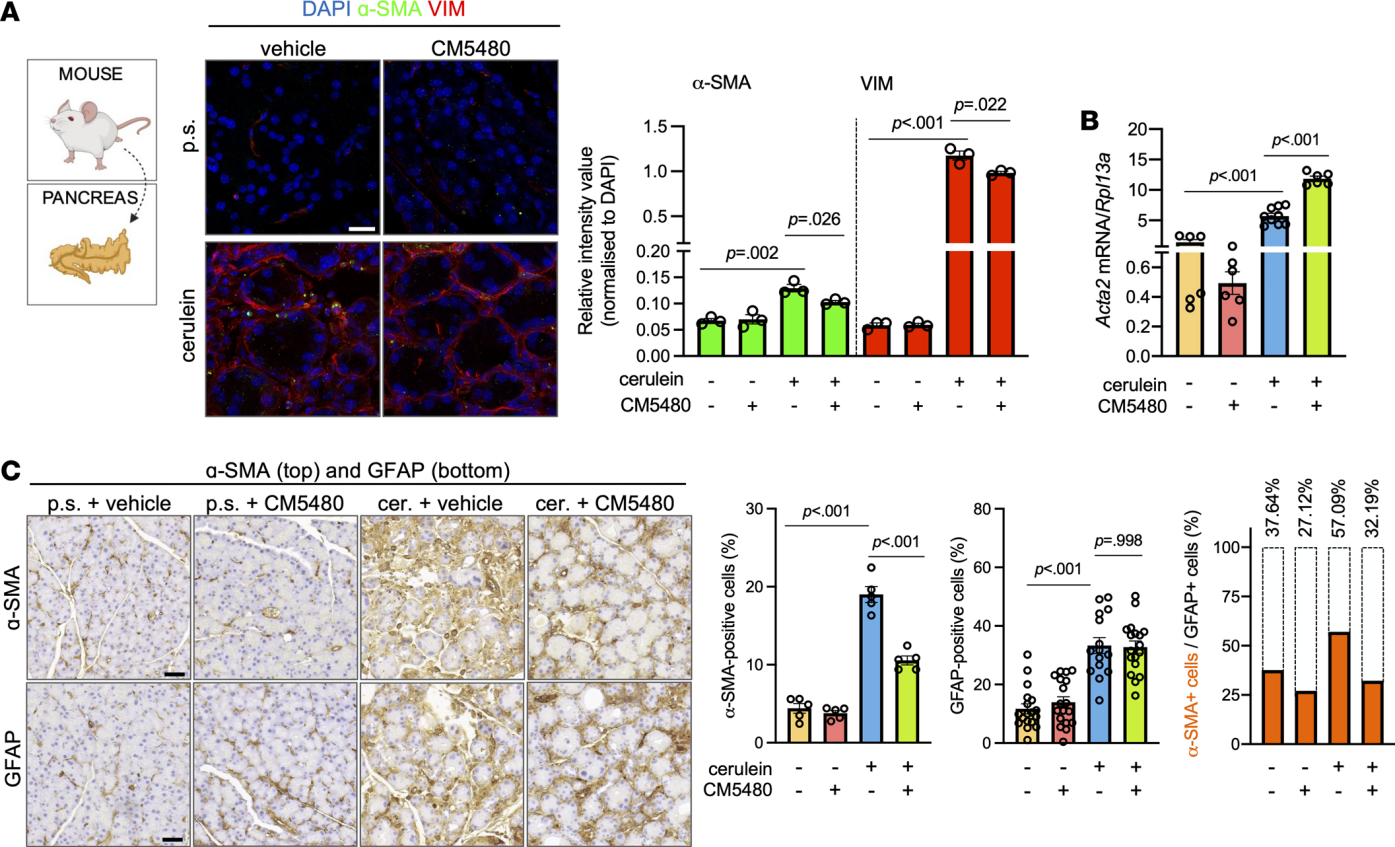
Orai1 inhibition impairs the activation of PSCs in CP. (**A**) Immunofluorescence staining and relative intensity values of α-SMA and VIM in mouse pancreatic tissue (*n* = 3/group, 5–7 images/animal). Scale bar: 20 μm. (**B**) *Acta2* gene expression in pancreatic tissue (*n* = 3/group). (**C**) IHC images of α-SMA (top) and GFAP (bottom) in mouse pancreatic tissues (*n* = 3/group, 5–7 images/animal). Scale bar: 50 μm. Bar charts summarize the ratio of α-SMA^+^ and GFAP^+^ and GFAP/α-SMA–double-positive cells (activated PSCs) (orange bars) in percentage of GFAP^+^ total cell number (bars with dashed line). A *P* value less than 0.05 was considered significant by unpaired *t* test (**A**) and Mann-Whitney *U* test (**B** and **C**). Data represent mean ± SEM. Explanatory images were created with BioRender. cer., cerulein; p.s., physiological saline.

**Figure 9:**
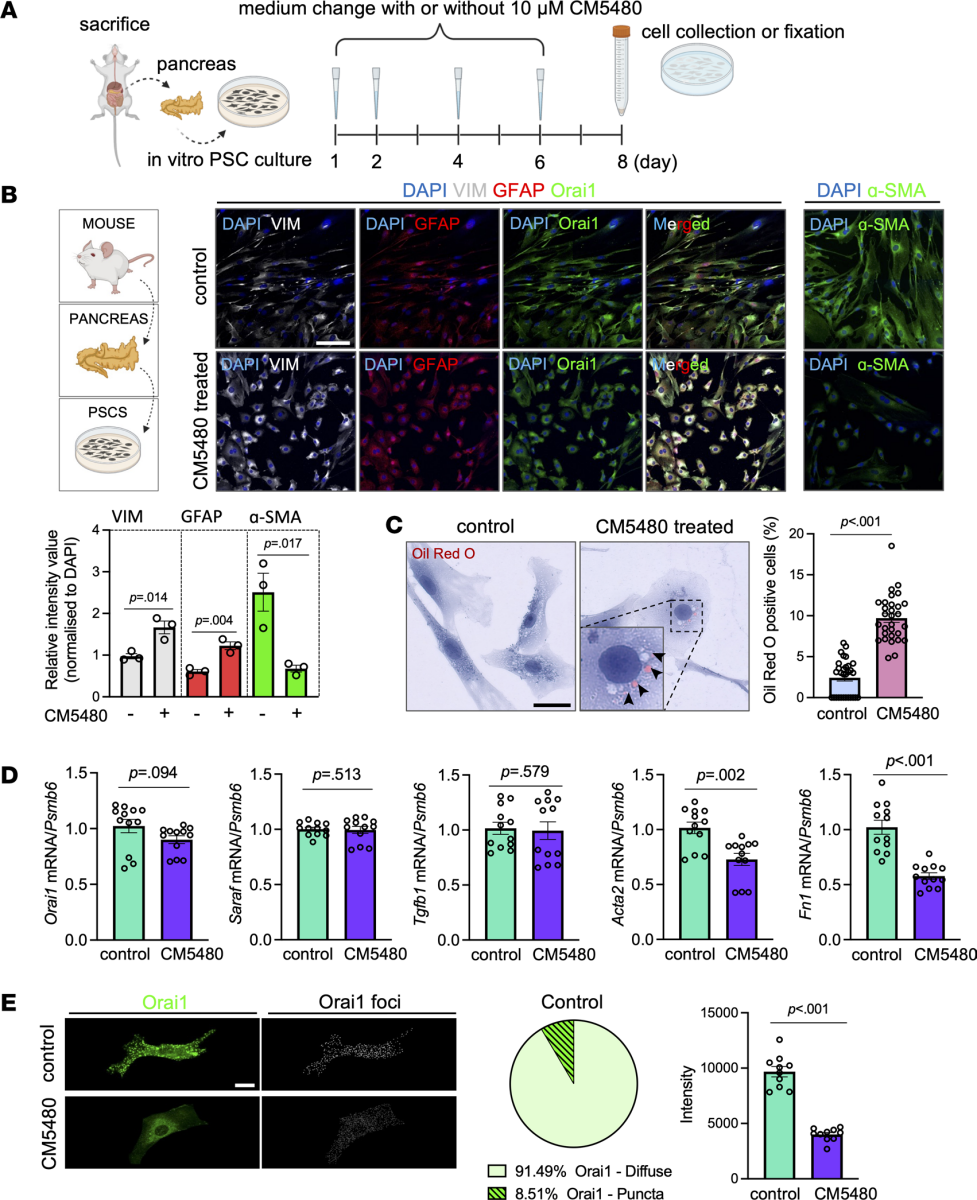
Activation of PSCs impaired upon CM5480 treatment in vitro. (**A**) Representative scheme of in vitro culturing of mouse PSC culture marking the CM5480 treatments during the 1-week time course. (**B**) Immunofluorescence staining and relative intensity values of VIM, Orai1, GFAP, and α-SMA in cultured PSCs. Scale bar: 100 μm. (**C**) Representative images and ratio of Oil Red O^+^ cells in cultured PSCs. *n* = 4/group, 5–7 images/culture, data points in the bars indicate individual field of views (**B** and **C**). Scale bar: 20 μm. (**D**) mRNA expression of *Orai1*, *Acta2*, *Tgfb1*, *Saraf*, and *Fn1* in cultured PSCs (*n* = 4/group from 4 animals). (**E**) Representative images and relative intensity values of Orai1 and percentage of PSCs with spontaneous Orai1 puncta formation in the control culture (*n* = 4/group, 5–7 images/culture). Scale bar: 10 μm. A *P* value less than 0.05 was considered significant by unpaired *t* test (**B** and **D** [*Orai1, Fn1*], and **E**) and Mann-Whitney *U* test (**C** and **D** [*Saraf, Tgfb1, Acta2*]). Data represent mean ± SEM. Explanatory images were created with BioRender.

To understand how Orai1 inhibition decreased pancreatic fibrosis in CP, we investigated the effect of CM5480 on PSCs. Immunofluorescence staining with the stellate cell markers α–smooth muscle actin (α-SMA) and vimentin (VIM) showed relatively low levels in the control pancreas, and the levels of these markers were significantly increased by cerulein administration (Figure 8A and Supplemental Figure 6A), and double-positive cells (activated PSCs) clearly showed periacinar localization. Orai1 inhibition significantly reduced the relative intensities of α-SMA and VIM, indicating a decrease of activated PSCs. On the other hand, *Acta2* (the gene encoding α-SMA) mRNA expression, which was elevated in the cerulein-treated group, was not decreased by CM5480 (Figure 8B). The decrease of α-SMA protein expression by Orai1 inhibition was also confirmed by IHC (Figure 8C and Supplemental Figure 6, B and C). Additionally, immunostaining of glial fibrillary acidic protein (GFAP), a pan-PSC marker (stains the quiescent and activated PSCs) revealed that PSCs were present in the control groups in a relatively low percentage but were increased after cerulein treatment. Notably, the increase in GFAP was not prevented by Orai1 inhibition. In contrast, the ratio of GFAP^+^ and α-SMA^+^ PSCs indicated that activated PSCs were dominantly present in CP and were remarkably diminished by CM5480. PSCs are known to activate rapidly in in vitro culture (within 48 hours) (24), which was utilized to study the effect of Orai1 inhibition on the activation process. Therefore, PSCs were isolated from control mice and maintained in vitro up to 1 week (Figure 9A). PSCs were treated with 10 μM CM5480 or vehicle every other day for 1 week and were immunostained for VIM, GFAP, α-SMA, and Orai1. The relative intensities of VIM and GFAP were lower, while the intensity of α-SMA was higher in the control group, indicating the activation of PSCs in the control sample (Figure 9B). In contrast, CM5480 treatment increased VIM and GFAP but decreased the α-SMA expression, suggesting inhibition of the PSC activation. Additionally, Oil Red O staining for Vitamin A revealed that the lipid droplets within the PSCs were relatively rare in the myofibroblast-like cells in the control culture, while CM5480 treatment significantly increased the percentage of the round-shaped lipid droplet containing quiescent PSCs (Figure 9C and Supplemental Figure 7A). Gene expression analysis showed no change of *Orai1*, *Saraf*, and *Tgfb1* mRNA levels, while *Acta2* and Fibronectin-1 (*Fn1*) expression, which are markers of activated PSCs (24, 25), significantly reduced upon Orai1 inhibition (Figure 9D). Notably, activated myofibroblast-like PSCs in the control culture displayed spontaneous Orai1 puncta formation (8.51% ± 1.373% of the cells), a well-described feature of Orai1 activation (Figure 9E), which was accompanied by extracellular Ca^2+^ influx (Supplemental Figure 7B). Such cells were not detected in the CM5480-treated cultures. In addition, the intensity of Orai1 was significantly higher in the control, compared with the CM5480-treated cells. These results demonstrate that inhibition of Orai1 prevented the transformation of quiescent-to-activated PSCs, which can explain the significantly impaired progression of fibrosis in CP.

### Orai1 inhibition decreases the proliferation and impairs the migration of activated PSCs.

**Figure 10:**
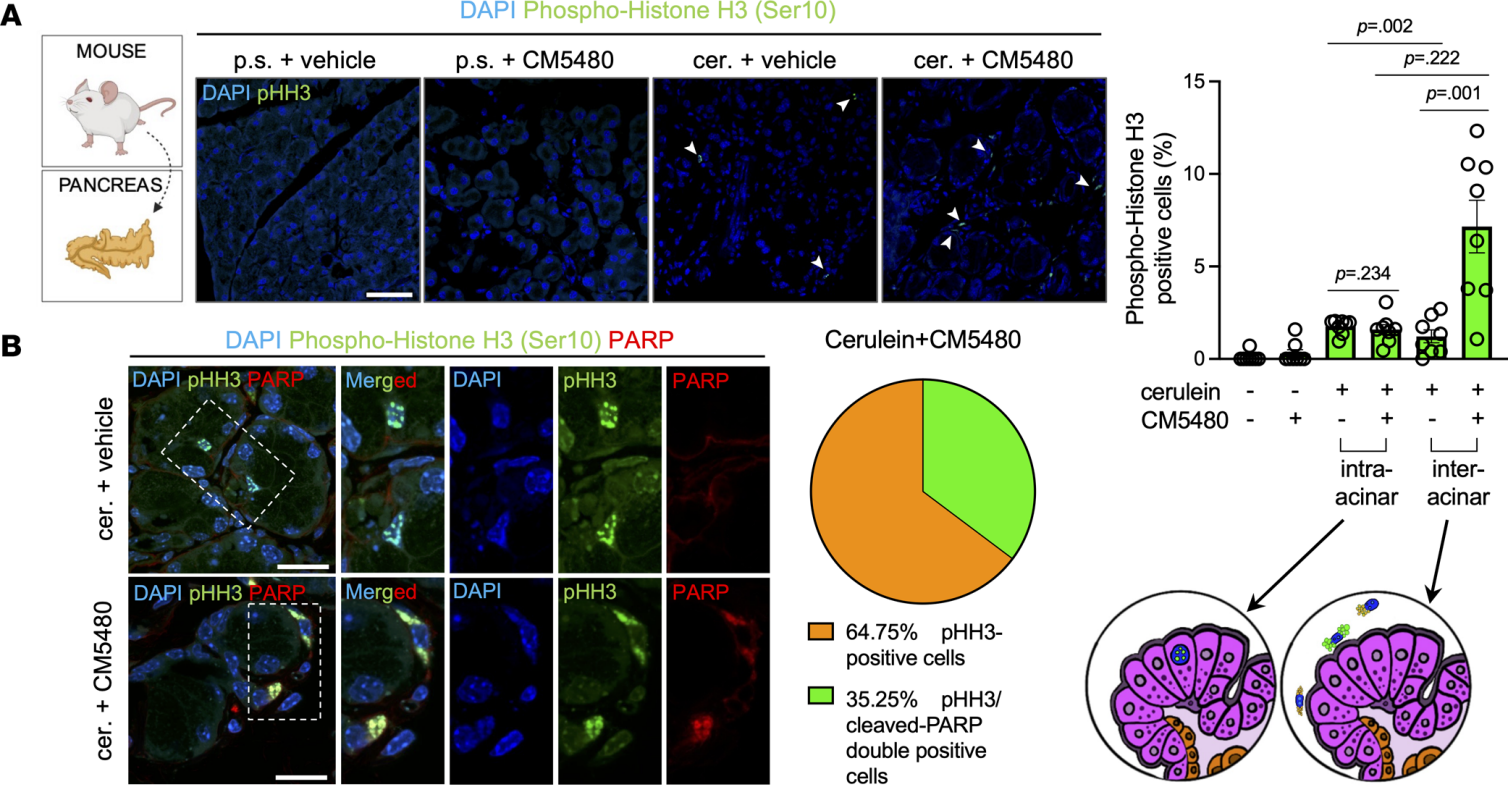
Orai1 inhibition increased the amount of pHH3 and cleaved PARP–double-positive cells. (**A**) Immunofluorescence staining and percentage of phospho–Histone H3 (Ser10)^+^ (pHH3^+^) cells in mouse pancreatic tissue. White arrowheads indicate the pHH3^+^ cells. Scale bar: 50 μm. (**B**) Immunofluorescence staining of pHH3 and PARP in mouse pancreatic tissues. *n* = 3/group, 5–7 images/animal (**A** and **B**). Scale bar: 50 μm. A *P* value less than 0.05 was considered significant by Mann-Whitney *U* test (**A**). Data represent mean ± SEM. Explanatory images were created with BioRender and ProCreate. cer., cerulein; p.s., physiological saline.

**Figure 11:**
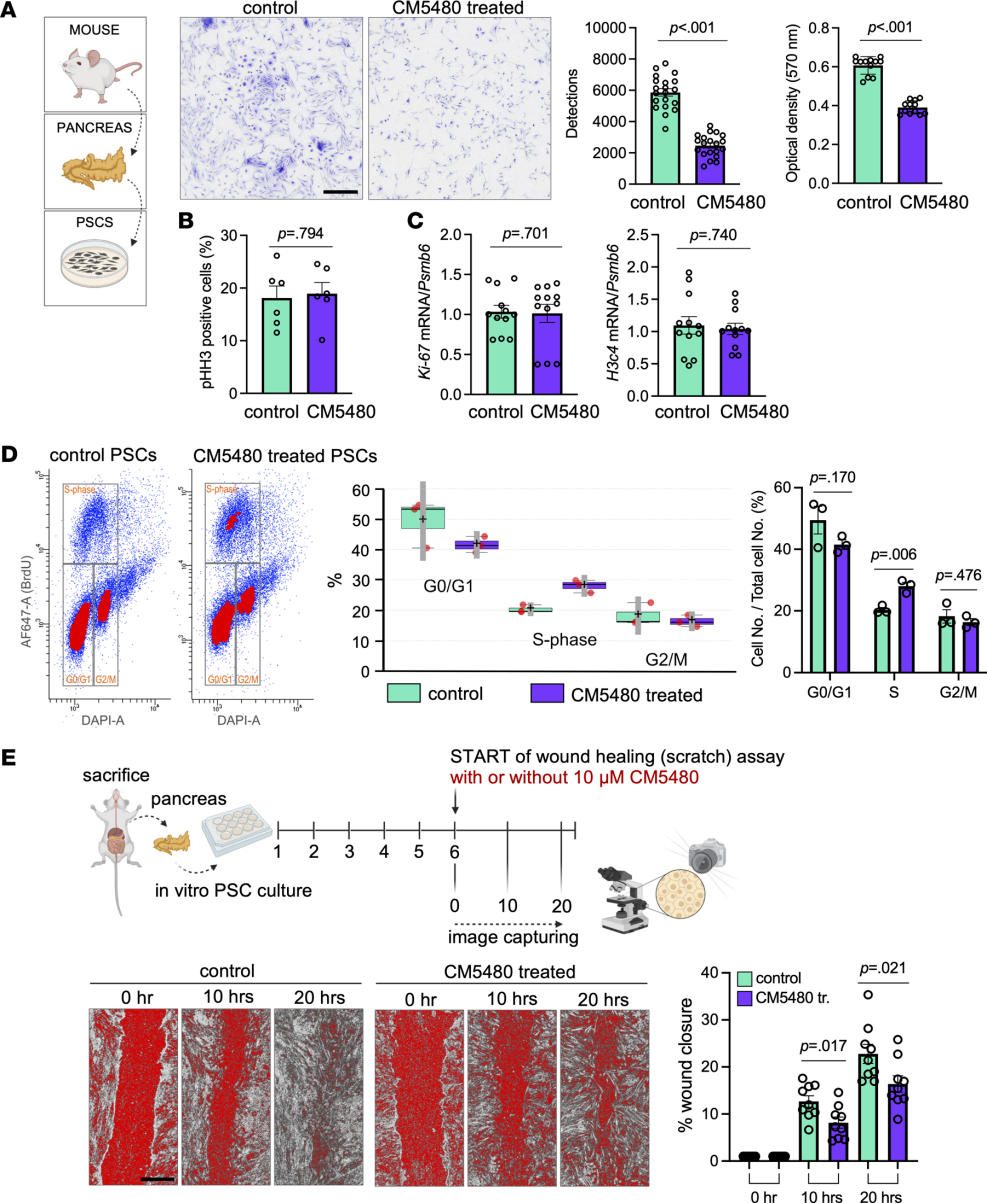
Orai1 inhibition impairs the migration and proliferation of PSCs. (**A**) Crystal Violet staining of cultured PSCs. Bar graphs indicate the number of cells and optical density of solubilized Crystal Violet. Scale bar: 50 μm. (**B**) Ratio of pHH3^+^ cells in cultured PSCs. (**C**) mRNA expression of *Ki-67* and H3 Clustered Histone 4 (*H3c4*). (**D**) Scatter diagrams indicate distribution of cell cycle phases. Contour plot/density coloring shows higher intensity values as red. Bar graph and box plot indicate the ratio of cell cycle phases of 3 replicates. (**E**) Representative scheme of in vitro wound healing (scratch) assay of mouse PSC culture marking the key experimental time points. Representative images and graph show the percentage of wound closure after 0, 10, and 20 hours. Scale bar: 500 μm. For **A** and **B**: *n* = 5/group from 4 animals; for **C**: *n* = 4/group from 4 animals; for **D**: *n* =3/group from 3 animals; for **E**: *n* =2/group from 9 animals. Data points in the bars represent individual cultures. A *P* value less than 0.05 was considered significant by unpaired *t* test (**A** [*Detections*], **B** and **C** [*H3c4*], **D** and **E**) and Mann-Whitney test (**A** [*Optical density*], **C** [*Ki-67*]). Data represent mean ± SEM. Explanatory images were created with BioRender. PSCs, pancreatic stellate cells; tr., treated.

Activation of PSCs greatly increases their proliferation and migration (26). To investigate the effect of Orai1 inhibition to these parameters, mouse pancreatic tissue was immunostained for the mitotic marker phospho–Histone H3 (Ser10) (pHH3) (Figure 10A and Supplemental Figure 8). While pHH3^+^ cells were only sporadically detected in the control groups, cerulein treatment significantly increased the number of proliferating cells within the acinar clusters (intraacinar) and in the inter- or periacinar space. Notably, significantly more proliferating cells were observed in the interacinar areas in the cerulein + CM5480–treated group (Figure 10B). Since pHH3 also marks apoptotic cells (27), cerulein + vehicle and cerulein + CM5480 pancreatic tissues were costained for pHH3 and cleaved PARP (a marker of apoptosis). Double-positive cells were absent in cerulein-treated samples but were present in the inter-/periacinar area of CM5480-treated group (35.25% ± 0.83%) beside the pHH3 single-positive cells. For further analysis, PSCs were isolated and cultured in vitro. First, the number of PSCs in culture was assessed by staining with Crystal Violet, which showed a significantly lower cell number in the CM5480-treated samples based on image analysis and colorimetric measurement as well (Figure 11A and Supplemental Figure 9, A and B). In vitro pHH3 staining was performed to determine whether the decreased cell number was due to increased apoptosis or decreased proliferation, but no significant difference was found between the control and CM5480-treated cultures (Figure 11B and Supplemental Figure 10). Similarly, no difference was observed in the gene expression of the proliferation marker *Ki-67* and H3 clustered histone 4 (*H3c4*), a core component of the nucleosome (Figure 11C). Next, we quantified the percentage of BrdU^+^ cells (marking the newly synthetized DNA) with flow cytometry, which showed that the number of cells in the S phase was significantly higher in the CM5480-treated sample as compared with control. Concomitant with this increase, we have also observed a decrease of average number of cells in G1 and G2/M phases in the treated cultures (Figure 11D and Supplemental Figure 11). These results indicate that the S to G2/M transition was slowed or blocked by the inhibition of Orai1, pointing to the importance of SOCE in this cell cycle phase in PSCs. Another plausible explanation for the lower cell number in the Orai1-inhibited sample could be an increased rate of cell death. This was further supported by the increased number of PARP^+^ PSCs in vivo, indicative of enhanced apoptosis. To test this, the late apoptosis/necrosis and living cell ratio was determined, and it showed a moderate decrease of living cells and an increase of apoptotic cells in the CM5480-treated group; however, the difference may not be biologically relevant (Supplemental Figure 12A). The gene expression of the late apoptotic marker *Casp3* was moderately lower in the CM5480-treated cell cultures (Supplemental Figure 12B), whereas PARP staining showed no difference (Supplemental Figure 12C). Altogether, these data suggest that the lower cell number in the CM5480-treated samples was caused by the impaired proliferation and not by increased cell death. Finally, the migration of PSCs was compared by determining the wound healing at 0, 10, and 20 hours (Figure 11E and Supplemental Figure 13). The images showed that the inhibition of Orai1 significantly impaired the speed of PSC migration, since the percentage of wound closure was significantly lower after 10 and 20 hours of recovery. In summary, we can conclude that inhibition of Orai1 significantly impairs the S to G2/M transition, cell proliferation, and migration of PSCs.

## Discussion

In this study, we demonstrate that SARAF expression is remarkably impaired in pancreatic acinar cells of mice and humans with CP, which can contribute to the excess extracellular Ca^2+^ entry. Furthermore, inhibition of Orai1 diminished the uncontrolled Ca^2+^ influx, restored SARAF expression, and prevented the progression of early CP to end-stage disease. This improvement was achieved by impaired inflammatory cell infiltration, protected acinar enzyme production, maintained ductal secretory function, and prevented activation of PSCs, which decreased tissue damage and fibrosis. Thus, we suggest that Orai1 inhibition could be the first specific therapy to prevent the development of end-stage CP.

Genetic and environmental factors, such as alcohol consumption and cigarette smoking, can damage pancreatic acinar and ductal functions (28, 29), which will induce the activation of quiescent PSCs into α-SMA^+^ myofibroblast-like phenotype producing excessive amount of extracellular matrix (ECM) leading to fibrosis (30). Aberrant intracellular Ca^2+^ signaling, which is well established in AP, plays a central role in chronic inflammatory processes as well; therefore, it could be a universal drug target. Our results show that the expression of SARAF, an ER membrane protein that mediates slow Ca^2+^-dependent inactivation of Orai1 (10), is remarkably decreased in pancreatic acinar cells during CP both in mice and humans, accompanied by increased extracellular Ca^2+^ influx. This excess Ca^2+^ influx was significantly impaired by selective Orai1 inhibition. In a recent paper, Son et al. described that the protein and gene expression of SARAF decreased during AP, whereas *Saraf*-KO mice developed more severe AP accompanied by increased Ca^2+^ influx in acinar cells (14). Previous studies demonstrate that sustained intracellular Ca^2+^ overload leads to premature activation of trypsinogen in pancreatic acinar cells (31) as well as mitochondrial damage and cell necrosis in pancreatic ductal cells. Previously, Gerasimenko et al. showed that the CRAC channel blocker developed by GlaxoSmithKline, GSK-7975A, inhibited SOCE in a concentration-dependent manner in pancreatic acinar cells (16). They also showed that the inhibition of CRAC channels impaired the sustained intracellular Ca^2+^ elevation induced by palmitoleic acid ethyl ester (a nonoxidative ethanol metabolite; ref. 32), whereas trypsin and protease activity as well as necrosis were almost abolished by GSK-7975A. In another study, Wen et al. showed that the inhibition of Orai1 decreased the severity of experimental AP in mice (15). Our group recently provided evidence that the administration of CM5480 prevented the development of sustained intracellular Ca^2+^ increase in pancreatic ductal cells during AP that protected the ductal ion and fluid secretion (17). Furthermore a recent manuscript suggests that the inhibition of Orai1 led to impaired inflammatory cytokine expression and prevented oxidative burst in neutrophils, leading to an improvement in the severity of AP in mice (33). On the other hand, Ahuja et al. report that Orai1 has a prominent role of the secretion of cathelicidin-related antimicrobial peptide by pancreatic acinar cells (34). Deletion of *Orai1* in the pancreatic acini of adult mice resulted in dysbiosis, systemic translocation, and increased mortality. Importantly, our results reveal that the inhibition of Orai1 prevented the drop of SARAF expression, diminished parenchyma atrophy, and interrupted the progression of fibrosis during early CP in mice. The fact that Orai1 inhibition prevents the drop of SARAF expression may suggest that the overactivation of Orai1 is triggered earlier. This is followed by the damage of SARAF expression, which can further augment the influx of extracellular Ca^2+^ as a vicious cycle. However, we cannot completely rule out the effect of other indirect mechanisms on SARAF expression, such as the impaired inflammatory response in the presence of CM5480. The clarification of this mechanism requires further analysis. These results suggest that the Orai1-mediated excess Ca^2+^ influx may play a significant role in the pathogenesis of RAP and the development of CP, whereas selective Orai1 inhibition has substantial benefits in CP by preventing the progression of early CP into the end-stage disease.

In the next steps, we comprehensively analyzed the pathophysiological role of Orai1 in the different cells of the exocrine pancreas. Inflammatory cells — predominantly mononuclear cells (mostly macrophages) — play a central role in the initiation and progression of CP (35), whereas the central role of Orai1 in the activation of T lymphocytes is well known (36). In alignment with this, our results show that Orai1 inhibition impaired the gene expression of *Tnfa* and *Il1b* (2 pleiotropic cytokines having an essential role in the pathogenesis of cerulein-induced pancreatitis; ref. 37) and *Tgfb1* (a critical regulator of PSC activation and fibrosis; ref. 38). Moreover, Orai1 inhibition also impaired the infiltration of different immune cells into the pancreatic tissue. Furthermore, when we comprehensively analyzed the exocrine pancreatic functions, we observed similar beneficial effects of Orai1 in CP compared with those reported in AP. The administration of CM5480 was sufficient to maintain the production of digestive enzymes in pancreatic acinar cells of CP mice, since both tissue amylase and elastase activities were significantly higher in this group. Pancreatic ductal cells secrete a large volume of HCO_3_^–^-rich alkaline fluid that flushes digestive enzymes from the ductal tree and prevents the autoactivation of trypsinogen (39). The proper ductal cell function largely depends on the activity of the CFTR Cl^–^ channel, whereas dysfunctional secretion and impaired apical plasma membrane localization of CFTR (23) in CP leads to formation of protein plugs, upstream ductal obstruction, and gland atrophy (40). Moreover, sustained elevation of intracellular Ca^2+^ diminishes the fluid and HCO_3_^–^ secretion from ductal cells (41). Our group previously published that Orai1 inhibition prevents the impairment of ductal cell secretion in bile acid or alcohol-induced AP (17). In the current study, we show that repetitive episodes of RAP diminished ductal HCO_3_^–^ secretion and CFTR function and that, in addition, CFTR localization was shifted from the plasma membrane to the cytosol, leading to impaired apical expression of CFTR. Orai1 inhibition prevented the decrements in ductal secretory functions and CFTR activity, and the apical membrane expression of CFTR was recovered. Importantly, the in vivo fluid secretion of the exocrine pancreas was maintained by the inhibition of Orai1 during CP.

In the healthy pancreas, quiescent PSCs (containing vitamin A–storing lipid droplets and expressing GFAP, desmin, and VIM) maintain the normal tissue architecture and regulate the synthesis and degradation of ECM proteins (42, 43). In CP, α-SMA^+^ myofibroblast-like PSCs secrete excessive amounts of ECM proteins, leading to fibrosis (24). Gryshchenko et al. demonstrated that bradykinin (BK)-elicited Ca^2+^ signals were caused by Ca^2+^ release from inositol triphosphate sensitive stores, followed by Ca^2+^ entry through CRAC channels such as Orai1 (44), a mechanism that can lead to PSC activation and proliferation in AP (45). Our results reveal that the administration of cerulein increased the activation of PSCs, which was significantly decreased by administration of CM5480. This was further tested in vitro utilizing isolated PSCs, which are known to activate almost immediately on an artificial surface (30). In accordance with this, untreated PSCs were positive for α-SMA, while cells treated with CM5480 displayed higher GFAP and VIM expression, displayed lower expression of *Acta2* and *Fn1* genes, and carried vitamin A–containing lipid droplets. Additionally, Orai1 protein formed puncta accompanied by extracellular Ca^2+^ influx in the control cultures, suggesting an active SOCE mechanism, which was not observed in the CM5480-treated cultures. These results suggest that the inhibition of Orai1 is sufficient to hinder PSC activation in CP. Other hallmarks of activated PSCs are high proliferation and migration activity, which also contribute to the enhanced fibrosis in CP (42). Our experiments revealed an increased number of pHH3^+^ cells reflecting mitotic activity in cerulein-treated mice that was further increased by CM5480 treatment. Notably, the pHH3^+^ PSCs in CM5480-treated animals were also positive for the apoptotic marker PARP, suggesting increased apoptosis, which may explain the reduced fibrosis. In HeLa cells exposed to apoptotic stimuli, activated PKCδ phosphorylates HH3 to facilitate chromatin condensation during apoptosis (27); this may explain the presence of pHH3/PARP–double-positive cells. The difference in the number of PSCs between the control and CM5480-treated samples was evident under in vitro conditions; however, the proliferation and apoptotic markers showed no difference, which could be explained by the lack of the complex intercellular signaling niche, such as the effect of acini on PSCs (46). These results suggest the possibility that, although pHH3^+^ cells enter mitosis, they are arrested in one of the cell cycle phases leading to impaired proliferation. Indeed, flow cytometry revealed that the PSCs treated with CM5480 are present in the S phase in a significantly higher percentage compared with the control. Finally, migration is also a key activation parameter of PSCs (26), and it was significantly impaired by Orai1 inhibition.

In the pathogenesis of CP, 5 main mechanisms have been hypothesized. First, a necrosis-fibrosis sequence of CP has been hypothesized that develops through episodes of severe AP leading to PSC activation and fibrosis (47). Second, the sentinel AP event, in which a single episode of AP causes inflammatory cell infiltration and PSC activation (47). Third, the direct toxic effect of environmental factors and their metabolites on acinar cells contribute to CP (48). Fourth, oxidative stress due to free radicals results in membrane lipid oxidation, inflammation, and fibrosis (49). Fifth, a ductal cell dysfunction leads to the formation of protein plugs and upstream ductal obstruction (50). Based on these mechanisms, the data presented in our manuscript have significant translational potential, as we were able to address all 5 current mechanisms by the inhibition of Orai1. Notably, Auxora — a selective Orai1 inhibitor — was tested in a phase 2 clinical study for patients with AP, SIRS, and hypoxemia; improved moderate AP in 36.5% of the patients; and enhanced the tolerance of solid food (51). Auxora is an i.v. drug that is not suitable for chronic administration; however, the development of an oral formulation could be used to treat patients with RAP and early CP. Obviously, the evaluation of immune functions during the chronic administration of Orai1 inhibitors should be an overall priority and must be carefully assessed by clinical trials.

Our study has some potential limitations. First, whether there is a suitable murine model of human CP currently remains unclear (52). In our study, the used in vivo disease model displayed the crucial features of CP; therefore, we utilized it in this proof-of-concept study. Notably, we have no information about the tissue regeneration in this mouse strain after the induction of CP. In the experimental protocol, the mice were sacrificed in the same time points; therefore, the beneficial effects of Orai1 inhibition could not be explained by the spontaneous tissue regeneration. Further studies will be needed to properly address this issue. Another unmet clinical need in the field is the prevention of the development of pancreatitis-induced diabetes (2). Therefore, analyses of the potential impact of Orai1 inhibition in the development of endocrine insufficiency in CP has to be assessed in further studies.

Taken together, in this study, we demonstrate that Orai1 inhibition prevents the progression of RAP and early CP into established CP. We show that CM5480 restores SARAF expression in acinar cells, prevents uncontrolled Ca^2+^ elevation, protects acinar and ductal functions, impairs immune cell infiltration, and diminishes PSC activation, proliferation, and migration. These results support the initiation of clinical studies to assess the beneficial effects of Orai1 inhibition in patients with RAP and early CP.

## Methods

All materials, antibodies, primers, and solutions used in the study are listed in Supplemental Tables 1–15. The list of commonly used materials and tools is summarized in Supplemental Tables 1–4. Detailed protocols are provided in Supplemental Information.

### Human samples.

Human pancreatic tissue samples (from control and patients with CP) were obtained from formalin-fixed paraffin-embedded (FFPE) tissue samples collected at the Department of Pathology, University of Szeged. The diagnosis of CP was established based on the clinical history and histological findings. Control pancreas samples were obtained from cadaver organ donors, who had no documented pancreatic disease.

### Animals.

Eight- to 12-week-old FVB/N mice weighing 20–25 g were utilized in our experiments. The mice were kept at a constant room temperature of 22°C–24°C with a 12-hour light/dark cycle and free access to food and drink. The sex ratio was 1:1. VRF1(P) standard rodent food and standard bedding were used. The treatments were carried out during the light cycle.

### Preparation of CM5480 for experimental use.

The Orai1 inhibitor compound is a dry powder that was manufactured and provided by CalciMedica. For i.p. dosing, the compound was dissolved in a vehicle containing 5% (2-Hydroxypropyl)-β-cyclodextrin (HPβCD) (w/v)/75% PEG400 (v/v)/20% water (v/v). The stock solution contained 4 mg/mL CM5480; therefore, to attain 20 mg/kg (i.e., 5 mL/kg) dosing for a 25 g mouse, 125 μL of formulated material was injected. For in vitro treatment of PSCs, CM5480 was dissolved in DMSO preparing a 100 mM stock solution and was used in 10 μM working concentration on cell cultures.

### Induction and evaluation of recurrent acute and CP.

RAP and CP was induced by repetitive i.p. cerulein injections. The mice received 3 or 5 series of 8 hourly physiological saline (p.s.; control group) or 50 μg/bwkg cerulein injections every third day (Figure 1C and Figure 3A). The selective Orai1 inhibitor CM5480 (i.p.; 20 mg/bwkg; provided by CalciMedica) or vehicle was administered daily on the last 5 consecutive days (after 3 episodes of RAP) (Figure 2B and Figure 3A). Twenty-four hours after the last cerulein injections, the mice were anesthetized and in vivo pancreatic fluid secretion was measured as described previously (32). The severity of CP was assessed by determining the pancreas weight/body weight ratio, histological parameters, and biochemical assays. Briefly, Crossmon’s trichrome staining was used to quantify the extent of fibrosis. Pancreatic tissue amylase and elastase activity was measured using an α-amylase kit (EPS) and EnzCheck Elastase Assay Kit, respectively. Protein concentration was measured by the Bradford method. Relative enzyme activity was normalized to the applied total protein concentration. HyP concentration was determined by using the Hydroxyproline Assay Kit and the amount of HyP (μg) were normalized to mg wet weight pancreas. All assays were performed according to the provided protocols with a CLARIOstar *Plus* plate reader (BMG Labtech).

### Isolation of pancreatic ductal fragments, acinar cells, and stellate cells from mouse pancreas.

Pancreatic ductal fragments were isolated as described earlier (29). Briefly, after terminal anesthesia, the pancreas was removed and digested with collagenase; then, intra/interlobular ducts were isolated under stereomicroscope. For pancreatic acinar and stellate cell isolation, the pancreas was enzymatically digested, minced into 1–3 mm^3^ pieces, and placed into shaking water bath at 37°C for 20 minutes (acinar) or 45 minutes (PSC) as described previously with modifications (32, 53). Acinar cells were collected and resuspended in supplemented Media 199 and used within 4 hours as previously described (32). After centrifugation (4°C at 110 x g for 10 minutes) and filtration of PSCs, the pellet was plated with feeding media. The medium was changed every other day. PSC cultures were maintained for 1 week until subsequent analysis (Figure 9A). Applied solutions for PSC isolation and maintenance are listed in Supplemental Tables 5–8.

### Measurement of intracellular Ca^2+^, Cl^–^, and pH by fluorescence microscopy.

Intracellular Ca^2+^ ([Ca^2+^]_i_) and Cl^–^ ([Cl]_i_) concentration, or intracellular pH (pH_i_), of isolated cells were evaluated by microfluorometry as described earlier by using Fura-2-AM (2 μmol/L), MQAE (2 μmol/L), or BCECF-AM (1 μmol/l) fluorescent dye, respectively (17, 54). The composition of solutions used during fluorescence measurements are listed in Supplemental Table 9.

### IHC and immunofluorescence labeling.

For histopathology or IHC pancreata were fixed in formalin and paraffin embedded, and 4 μm–thick sections were cut. For IHC, specific antibodies were used for immune cell markers (anti-CD3, Invitrogen, MA5-12577; anti-CD8, Invitrogen, PA588265; anti-CD19, Invitrogen, MA5-32560; anti-F4/80, Cell Signaling Technology, 70076; anti-Myeloperoxidase, Dako, A0398), SARAF (anti-TMEM66, Invitrogen, PA1-31588), α-SMA (anti–α-SMA, Cell Marque, 202M-94), GFAP (anti-GFAP, Bio SB, BSB5564), and α-amylase (anti-α-amylase, abcam, ab199132). Images were captured by Zeiss Axio Imager.M2 microscope (Carl Zeiss Microscopy) with 2.5***×*** objective (Zeiss, NA: 0.07), 5***×*** objective (Zeiss, NA: 0.15), 20***×*** objective (Zeiss, NA: 0.8), and 40***×*** objective (Zeiss, NA: 0.75); they were then subsequently analyzed. For immunofluorescence staining, whole samples were frozen and cut at –20°C; they were then fixed in 4% paraformaldehyde (PFA) or PSCs seeded onto cover glasses were fixed in 4% PFA and labeled with primary antibodies overnight (anti–α-SMA, Novus Biologicals, NB300-978; anti-CFTR, Alomone labs, ACL-006; anti-GFAP, Invitrogen, PA1-10004; anti-Occludin, Invitrogen, 33-1500; anti-Orai1, abcam, ab59330; anti-Orai1, Novus Biologicals, NBP1-75522; anti-PARP, Novus Biologicals, NB100-56599; anti-pHH3, Sigma-Aldrich, 06-570; anti-TMEM66, Invitrogen, PA5-31588; anti-VIM, Invitrogen, MA3745). Then, they were labeled with secondary antibodies for 2 hours (donkey anti–goat Alexa Fluor 488, Abcam, ab150129; donkey anti–mouse Alexa Fluor 647, Invitrogen, A31571; goat anti–mouse Alexa Fluor 488, Invitrogen, A48286; goat anti–mouse Alexa Fluor 555, Invitrogen, A48287; goat anti–mouse Alexa Fluor 647, Invitrogen, A48289; goat anti–rabbit Alexa Fluor 488, Invitrogen, A48282; goat anti–rabbit Alexa Fluor 647, Invitrogen, A48285; goat anti–chicken Alexa Fluor 647, Invitrogen, A32933). Images were captured with a Zeiss LSM880 confocal microscope (Carl Zeiss Microscopy) using a 40***×*** (Zeiss, NA: 1.4) and 63***×*** oil immersion objective (Zeiss, NA: 1.46). Antibodies used are listed in Supplemental Tables 10–13 in detail. The image analysis is detailed in the Supplemental Information.

### Gene expression analysis.

mRNA from human FFPE and mouse pancreas samples was isolated by NucleoSpin totalRNA FFPE XS kit or NucleoSpin RNA Plus kit according to the manufacturer’s protocol (22). mRNA from PSCs was isolated by NucleoZOL based on the manufacturer’s instructions. In total, 1 μg mRNA was reverse transcribed using iScript cDNA Synthesis Kit, and 50 ng cDNA was used for quantitative PCR (qPCR) analysis in a LightCycler 96 System (Roche). The used primers are listed in Supplemental Table 14. The fold change in transcript levels was calculated by normalizing the threshold values to *Psmb6* (to *Cftr* and in case of in vitro experiments), *GAPDH* (to *SARAF*), and *Rpl13a*.

### Western blot analysis.

Pancreatic tissues were sonicated in RIPA lysis buffer containing complete protease inhibitor cocktail and complete phosphatase inhibitor cocktail. Protein concentrations were determined by the BCA assay, and 20 μg of protein was loaded onto a 15% Bis-Tris gel and transferred to a PVDF membrane using the Bio-Rad PROTEAN system (Bio-Rad). Proteins were detected by probing with anti-TMEM66 (SARAF) (Invitrogen, PA5-31588) and anti–β-actin (Cell Signaling Technology, 4967S) followed by anti–rabbit HRP (goat anti–rabbit HRP conjugate, Invitrogen, 31460) and developed with Clarity Western ECL Substrate before being visualized on the ChemiDoc Imaging System (Bio-Rad). Antibody dilutions, solutions, and gel compositions are listed in Supplemental Tables 13 and 15.

### Oil Red O staining, in vitro proliferation, migration, and cell death measurements.

PSCs were plated into cell culture dishes and stained with Oil Red O solution. Slides were mounted with Fluoromount Aqueous Mounting Medium, and images were captured by Zeiss Axio Imager.M2 microscope. The cell densities were determined by Crystal Violet staining. Images were captured by Zeiss Primovert microscope (Carl Zeiss Microscopy) and evaluated by Fiji (build 1.53c Java version 1.8.0_172, NIH) and QuPath (version 0.3.0 Java version 16.0.2). Alternatively, Crystal Violet dye was solubilized, and optical density was measured with a CLARIOstar *Plus* plate reader. To analyze the cell cycle phase ratio, PSCs were incubated with BrdU for 1 hour and labeled with anti-BrdU antibody (anti-BrdU monoclonal antibody, Invitrogen, MA3-071) and DAPI; then, flow cytometry analysis was performed. The living/apoptotic/necrotic cell ratio was determined by Apoptosis/Necrosis Assay Kit in CLARIOstar *Plus* plate reader. For the wound-healing assay, PSCs were grown to confluency for 1 week, and a cell-free region was made by scratching the cell monolayer and monitor cell migration (Figure 11E). Images were taken at 0, 10, and 20 hours after scratch by Zeiss Primovert microscope and analyzed with Fiji software.

### Statistics.

Statistical analysis and strength of significance were performed with GraphPad Prism software. All data are expressed as mean ± SEM. Shapiro-Wilk normality test was applied. Both parametric (Unpaired, 2-tailed *t* test or 1-way ANOVA with Tukey’s multiple-comparison test) and nonparametric (Mann-Whitney *U* test and Kruskal-Wallis test) tests were used based on the normality of data distribution. A *P* value below 0.05 was considered statistically significant.

### Study approval.

The NIH rules and the EU directive 2010/63/EU were followed while using animals. The National Scientific Ethical Committee on Animal Experimentation granted the study license number XXI./1541/2020. The collection and use of human samples, including cadaver donor pancreas, was carried out in accordance with EU norms and was approved by the Hungarian Medical Research Council’s Regional Committee of Research Ethics under license number 37/2017-SZTE.

### Data availability.

The data sets generated during and/or analyzed during the current study are fully available upon contact with the corresponding author.

## Author contributions

For defining authorship, the recommendations of the International Committee of Medical Journal Editors were followed. The final author order was determined based on the individual contribution of the authors. VS, PP, KS, SH, and JM designed the research project. VS, NCP, MG, TM, ÁV, AK, BT, BJ, TC, KD, FA, and SJ contributed to acquisition, analysis, and interpretation of data for the work. MB and NB performed the histology investigations. LT provided human pancreatic tissue samples. VS, PP, and JM drafted the work, and all authors approved the final version of the manuscript and agreed to be accountable for all aspects of the work in ensuring that questions related to the accuracy or integrity of any part of the work are appropriately investigated and resolved. All persons designated as authors qualify for authorship, and all those who qualify for authorship are listed.

## Supplementary Material

Supplemental data

## Version 1 05/25/2023

In-Press Preview

## Version 2 07/10/2023

Electronic publication
