# Supplementary material for: Orai1 calcium channel inhibition prevents progression of chronic pancreatitis
Source: JCI Insight. 2023 Jul 10;8(13):e167645. doi: 10.1172/jci.insight.167645 (PMC10371343; doi:10.1172/jci.insight.167645)
Supplement: Supplemental data [file jciinsight-8-167645-s148.pdf]

Supplemental information

Supplemental materials and methods

The list of commonly used materials and tools is summarized in **Supplemental Table 1-4.**

Isolation of pancreatic ductal fragments and acinar cells

Pancreatic ductal fragments were isolated as described earlier (1). Briefly, animals were anaesthetized terminally by pentobarbital, the pancreas was surgically removed, put into ice-cold DMEM/F12 and injected with a digestion solution, which contain 100 U/ml collagenase, 0.1 mg/ml trypsin inhibitor, 1 mg/ml bovine serum albumin. After 30 min shaking at 37ºC, small intra- and interlobular ducts were isolated under a stereomicroscope by using microdissection. For pancreatic acinar cell isolation, the pancreas was quickly removed, placed into ice-cold HBSS and cleaned from fat and lymph nodes. Tissues were transferred to a freshly prepared digestive solution containing 200 U/ml type 4 collagenase, 10 mM HEPES and 0.25 mg/ml trypsin inhibitor, minced into 1-3 mm^3 pieces and placed into a shaking water bath at 37°C for 20 min as described previously (2). Tissue was washed with HBSS supplemented with 10 mM HEPES, 0.25 mg/ml soybean trypsin inhibitor and 5% FBS and centrifuged 3x for 2 min at 450 RCF. The filtered pellet was resuspended in Media 199 with 2.5% FBS and 0.25 mg/ml soybean trypsin inhibitor.

Pancreatic stellate cell isolation from mouse pancreas

8-12-week-old wild-type FVB/N mice were sacrificed, the pancreas was removed and transferred into HBSS containing Antimycotic-Antibiotic Solution (1v/v%) and Kanamycin Sulfate (1v/v%). The pancreas was washed with physiological NaCl solution and finely minced. Tissue pieces were incubated in digestion solution (**Supplemental Table 5, 6**) at 37°C for 45

min at 165 rpm, resuspending strongly in every 15 min. The cell suspension was centrifuged at 750 rpm for 10 min at 4°C. Pellet was washed with wash media (**Supplemental Table 5, 7**) at least twice (750 rpm 10 min), carefully and gently resuspended between each washing step. Pellet was resuspended in 1 ml wash media and filtered with a 100 µm filter and centrifuged again. Pellet was plated with feeding media (**Supplemental Table 8**) into the proper cell culture dishes or plates. Half of the samples were treated with 10 µM CM5480. The pancreatic stellate cell (PSC) culture was incubated for 24 hours in incubator (37°C, 5% CO_2), then the medium was removed together with the unattached cells and cell debris and fresh medium was added to the cells (half of the samples with CM5480). The medium was changed every second day thereafter for one week (half of the samples with CM5480).

Tissue amylase and elastase activity

Pancreatic tissue amylase and elastase activity were measured with a kinetics colorimetric and fluorometric method using an alpha-amylase kit (EPS) purchased from Diagnosticum Zrt. (Budapest, Hungary) and EnzCheck™ Elastase Assay Kit, respectively. 20 mg pancreatic tissue was measured, finely minced and sonicated in 1x RIPA lysis buffer for 10 seconds three times. In case of amylase assay, lysis buffer was supplemented by PhosSTOP phosphatase inhibitor cocktail and cOmplete ULTRA protease inhibitor cocktail. Between each sonication cycle, samples were left to incubate on ice for 10 seconds. The homogenized tissues were centrifuged at 3500 rpm at 4°C for 10 minutes then the supernatant was transferred into new centrifuge tubes. Protein concentration was measured by Bradford method then 200 ng protein was loaded to the microplate. Relative enzyme activity was normalized to the applied total protein concentration. Tissue elastase activity was calculated through the slope of the linear phase (slope of range) of the fluorescence resulting from the cleavage of the DQ™ elastin conjugate

supplied in the kit. The results are represented as the changes in relative fluorescence units per minute (ΔRFU). Pancreata of minimum five mice were used from each experimental group.

Determination of hydroxyproline concentration

Hydroxyproline (HyP) concentration was determined by an end-point colorimetric method using the Hydroxyproline Assay Kit. 20 mg pancreatic tissue was finely minced and homogenized in dH_2O. 12N HCl was added to the samples in 1:1 ratio and the samples were hydrolyzed for 3 hours at 120°C. Precipitates were removed by centrifugation with 10,000 g for 3 minutes at RT. 10 µl of clear supernatant were loaded into each sample well and dried at 60°C. Assays were performed according to the kit protocol. Absorbance was measured at 560 nm by a CLARIOstar® *Plus* plate reader (BMG Labtech, Ortenberg, Germany). Hydroxyproline content was normalized to the wet weight of pancreatic tissue. Pancreata of minimum six mice were used from each experimental group.

Measurement of in vivo pancreatic fluid secretion

After induction of CP, pancreatic fluid was collected in vivo directly before the sacrifice of the animals (2). Mice were anesthetized with 125 mg/bwkg ketamine/12.5 mg/bwkg xylazine i.p. and placed on a heated pad to maintain body temperature. After median laparotomy, 0.4 mm diameter needle connected to an infusion catheter was introduced into the common biliopancreatic duct across the duodenum and the bile duct was occluded with a microvessel clip. After 30 min secretin stimulation (0.75 Clinical Unit/kg, i.p), the pancreatic juice was collected and the secretory rate was calculated as µl/body weight g for 1 hour.

Measurement of intracellular Ca^{2+}, Cl^- and pH by fluorescent microscopy

Intracellular Ca^{2+} ($[Ca^{2+}]_i$) and Cl^- ($[Cl]_i$) concentration, or intracellular pH (pH_i) were evaluated by microfluorometry as described earlier by using Fura-2-AM (2 μmol/l), MQAE (2 μmol/l), or BCECF-AM (1 μmol/l) fluorescent dye, respectively (3). Ducts were attached to a poly-L-lysine-coated coverslip and mounted on an Olympus IX73 fluorescent microscope equipped with a CoolLED pE-4000 or CoolLED pE-340Fura illumination system (Andover, UK). Filter sets used for Fura2, MQAE and BCECF measurements were described previously (4). Hamamatsu ORCA Flash 4.0 V3 CMOS camera was used to capture fluorescent signals through a 20x oil immersion objective (Olympus; NA: 0.8) with a temporal resolution of 1 sec. Ratiometric image analysis was performed by Olympus excellence software. The composition of solutions used during fluorescent measurements are listed in **Supplemental Table 9**.

Crossmon's Trichrome staining

To evaluate the severity of CP histological parameters were monitored. After the quick removal of pancreata, the tissues were cleaned from fat and lymph nodes and put in 4% formaldehyde for fixation and stored at 4°C. Paraffin-embedded pancreas samples were sliced into 4 μm thick sections and stained with Crossmon's Trichrome. After deparaffination, slides were submerged into pre-warmed saturated picric-acid solution for 30 sec. After a 15 min washing step with tap water, nuclei were stained with alum hematoxylin for 5 min. To stain keratin, Orange G solution was applied for 3 min, then samples were washed with acetic acid (2 ml/dl). Fuchsin acid staining was performed for 3 min, then slides were washed with acetic acid. Samples were incubated in Phosphotungstic acid (PTA, 5 g/dl) solution for 2 min and after a washing step with acetic acid aniline blue staining was performed for 10 min. After a washing step with acetic acid, slides were rinsed with isopropyl alcohol, air-dried and mounted with Eukitt® Quick-hardening mounting medium.

Immunohistochemistry

4-µm-thick sections of FFPE human or mouse pancreas samples were used for immunohistochemistry (IHC). Immunohistochemical labelling was performed with a Leica Bond-MAX Fully Automated IHC and ISH Staining System. Briefly, deparaffinization step was carried out with Bond™ Dewax Solution at 72°C. After washing with Bond™ Wash Solution, epitope retrieval was carried out with Bond™ Epitope Retrieval Solution 2 at 100°C for 20 minutes at pH 9. After slides were washed with wash solution at 35°C, peroxidase blocking was performed by Novocastra Peroxidase Block for 5 minutes. Primary antibodies were diluted in Bond™ primary antibody diluent and incubated on samples for 20 minutes. Applied primary antibodies are listed in **Supplemental Table 10.** in details (anti-CD3, Invitrogen, MA5-12577; anti-CD8, Invitrogen, PA588265; anti-CD19, Invitrogen, MA5-32560; anti-F4/80, Cell Signaling Technology, 70076; anti-Myeloperoxidase, Dako, A0398; anti-TMEM66, Invitrogen, PA1-31588; anti-alpha-SMA, Cell Marque, 202M-94; anti-GFAP, Bio SB, BSB5564; anti-alpha-amylase, abcam, ab199132). For immunohistochemical labelling and visualization, Bond™ Polymer Refine Detection was applied for 8 minutes. Formaldehyde-fixed pancreatic tissues were embedded in paraffin for routine histology and 4 µm thick slices were stained with hematoxylin and eosin. Images were captured by Zeiss Axio Imager.M2 microscope (Carl Zeiss Microscopy, Göttingen, Germany) with 2.5x objective (Zeiss, NA: 0.07), 5x objective (Zeiss, NA: 0.15) 20x objective (Zeiss, NA: 0.8) and 40x objective (Zeiss, NA: 0.75) then subsequently analyzed.

Immunofluorescence staining

Mouse pancreas or isolated pancreatic ducts from each experimental group was fixed in 4% paraformaldehyde (PFA) for 2 hours at 4°C then washed three times in 1x DPBS and cryoprotected in 30% sucrose at 4°C overnight. Cryoprotected pancreatic tissues was

submerged in Shandon CryoMatrix and 7 μm thick sections were cut and placed on Superfrost slides at -20ºC. Untreated and CM5480 treated PSCs were seeded into 6-well tissue culture plates onto acid-wash treated glass coverslips and maintained for 1 week. Cells were washed with 1x DPBS and fixed in 4% PFA for 20 min. After washing in 1x TBS solution three times, samples were permeabilized in citrate-Tween 20 solution (0.001 M Sodium Citrate Buffer, pH 6.0 and 0.05 % Tween 20) in 95°C for 30 min in a rice cooker. Slides were transferred into ice-cold TBS for 10 min and washed additionally 3 times in TBS for 10 min. Samples were stored in 10% BSA-TBS for non-specific antigen blocking for 2 hours at 37°C and incubated with primary antibodies applied in 10% BSA-TBS for overnight at 4°C (anti-alpha-SMA, Novus Biologicals, NB300-978; anti-CFTR, Alomone labs, ACL-006; anti-GFAP, Invitrogen, PA1-10004; anti-Occludin, Invitrogen, 33-1500; anti-Orai1, abcam, ab59330; anti-Orai1, Novus Biologicals, NBP1-75522; anti-PARP, Novus Biologicals, NB100-56599; anti-pHH3, Sigma-Aldrich, 06-570; anti-TMEM66, Invitrogen, PA5-31588; anti-Vimentin, Invitrogen, MA3745). Samples were washed three times in TBS for 10 min and incubated with secondary antibodies for 2 hours at RT (Donkey Anti-Goat Alexa Fluor 488, abcam, ab150129; Donkey Anti-Mouse Alexa Fluor 647, Invitrogen, A31571; Goat Anti-Mouse Alexa Fluor 488, Invitrogen, A48286; Goat Anti-Mouse Alexa Fluor 555, Invitrogen, A48287; Goat Anti-Mouse Alexa Fluor 647, Invitrogen, A48289; Goat Anti-Rabbit Alexa Fluor 488, Invitrogen, A48282; Goat Anti-Rabbit Alexa Fluor 647, Invitrogen, A48285; Goat Anti-Chicken Alexa Fluor 647, Invitrogen, A32933). Detailed primary and secondary antibody lists can be found in **Supplemental Table 11, 12** and **13**. Slides were washed two times with TBS for 10 min. The nuclei of the cells were labelled with DAPI (1 μg/ml) for 30 min at RT. Samples were washed three times with TBS for 10 min. Finally, slides were mounted by Fluoromount™ Aqueous Mounting Medium. Images were captured with a Zeiss LSM880 confocal microscope (Carl Zeiss Microscopy,

Göttingen, Germany) using a 40x (Zeiss, NA: 1.4) and 63x (Zeiss, NA: 1.4) oil immersion objective.

Gene expression analysis

Total mRNA from human formalin-fixed paraffin-embedded pancreatic tissues and mouse pancreas was isolated by NucleoSpin totalRNA FFPE XS kit or NucleoSpin® RNA Plus kit according to the manufacturer's protocol with modifications, respectively (5). Total mRNA from PSC culture was isolated by using NucleoZOL based on the manufacturer's instructions. In case of human samples, FFPE tissue blocks were sectioned in 20 µm thickness by microtome and paraffin was manually removed from the sections to eliminate the de-paraffinization step. Samples were lysed at 56°C for 3 hours and at 50°C for 21 hours until the whole tissue was dissolved. RNA concentrations were measured with a Spectrophotometer (NanoDrop™ One, Thermo Fisher Scientific, Massachusetts, US). 1 µg of total mRNA was reverse transcribed to cDNA with iScript™ cDNA Synthesis Kit. 50 ng of the cDNA was used for quantitative real-time PCR (qPCR) analysis with SsoAdvanced™ Universal SYBR® Green Supermix using the LightCycler® System (Roche, Basel, Switzerland). The cDNAs were amplified with the primers listed in **Supplemental Table 14**. The qPCR reactions were carried out under the following conditions: 2 min at 95°C and 40 cycles of 95°C for 30 sec, 58°C for 30 sec, 72°C 14 sec, then the final cooling step 37°C 30 sec. Fluorescent dye intensity was detected after each cycle. The fold change was calculated by normalizing the threshold values to *GAPDH* in case of human samples, to *Rpl13a* and *Psmb6* in case of mouse pancreas and to *Psmb6* for PSCs. The non-template control sample was used for each PCR run to check the primer-dimer formation. The final relative gene expression ratios were calculated as ΔCt values (Ct values of the gene of interest versus Ct values of the control gene).

Western blot analysis

Pancreatic tissues were sonicated in RIPA lysis buffer containing complete protease inhibitor cocktail and complete phosphatase inhibitor cocktail. Protein concentrations were determined by BCA assay then 5xLaemmli buffer was added to the diluted samples and incubated for 5 min at 95°C. Samples were set on ice and 20 μg of protein was loaded onto a 15% Bis-Tris gel and transferred to a PVDF membrane using the Bio-Rad PROTEAN system (Bio-Rad, California, United States). Membrane was blocked in blocking buffer (1% BSA + 0.1% Tween20 in TBS; TBS-T) for 1 hour at 4°C. First, proteins were detected by probing with anti-beta-actin (Cell Signaling Technology, 4967S) overnight followed by anti-rabbit–HRP (Invitrogen, 31460) for 1 hour. Signal was developed with Clarity Western ECL Substrate before being visualized on the ChemiDoc Imaging System (Bio-Rad, California, United States) (the exposition time was 2 min). Membrane was washed three times with TBS-T then hybridized with anti–TMEM66 (SARAF) (Invitrogen, PA5-31588) overnight followed by anti-rabbit–HRP (Invitrogen, 31460) for 1 hour. Signal was developed with Clarity Western ECL Substrate before being visualized on the ChemiDoc Imaging System (Bio-Rad, California, United States) (the exposition time was 20 sec). Image colors were inverted to negative and the intensity of each lane analyzed in Fiji ImageJ (build 1.53c Java version 1.8.0_172, NIH, USA). Background intensity of the membrane was subtracted from the individual integrated density (intensity) values and represented as arbitrary values on graph. Dilutions of antibodies are listed in **Supplemental Table 13**. Solutions and gel compositions are listed in **Supplemental Table 15**.

Oil Red O staining of control and CM5480 treated mouse PSCs

Untreated and CM5480 treated PSCs were plated into 3.5 cm cell culture dishes onto acid-wash pre-treated glass coverslips and maintained for 1 week. Oil Red O staining was performed based

on manufacturer's instructions. The cell surface was washed with 1x DPBS once, then incubated in propylene glycol for 2 min. Slides were incubated in pre-warmed (60°C) Oil Red O solution for 15 min then differentiated in 85% propylene glycol for 1 min. Slides were rinsed twice in distilled water and incubated in Hematoxylin for 2 min, then rinsed thoroughly in tap water, rinsed in distilled water mounted with Fluoromount™ Aqueous Mounting Medium. Images was captured by Zeiss Axio Imager.M2 microscope (Carl Zeiss Microscopy, Göttingen, Germany) with 40x objective (Zeiss, NA: 0.75).

Wound healing assay of mouse PSCs

PSCs were seeded into 12-well plates for probing collective cell migration in two dimensions (6). Cells were growing for one week until they formed a monolayer. The wound was made by scratching the cell monolayer in a straight line with a sterile P-200 pipette tip. Then the medium was changed to a fresh feeding medium either with vehicle or with 10 μM CM5480. Photos were taken at 0, 10 and 20 hours after scratch by Zeiss Primovert microscope (Carl Zeiss Microscopy, Göttingen, Germany) with 4x objective (Zeiss, NA: 0.01) and analyzed with Fiji ImageJ software package (build 1.53c Java version 1.8.0_172, NIH, USA). Images were cropped in 1:1 ratio, applied and auto contrast, black and white and negative effect to increase the detection of cell borders. The analysis of the re-colorized image was the following in Fiji ImageJ software package (build 1.53c Java version 1.8.0_172, NIH, USA): find edges (process) → Gaussian blur with sigma (radius) 2.00 (process) → Adjust threshold with lower threshold level 0, upper threshold level 60 (image) → RGB measure (plugins) → Red Integrated Density data. Nine wound healing assays were performed from each experimental group. The workflow scheme is represented in **Figure 11E**.

Crystal Violet assay of mouse PSCs

Untreated and CM5480 treated PSCs were seeded into 6-well tissue culture plates and maintained for 1 week. Cells were washed with cold PBS and fixed with ice-cold methanol for 10 min (7). After methanol removal Crystal Violet solution (0.125 g Crystal Violet powder, 10 ml methanol, 40 ml water) was added and samples were incubated for 15 min at RT during gentle shaking. Samples were carefully washed with tap water three times and air-dried upside down. Photos were taken by Zeiss Primovert microscope (Carl Zeiss Microscopy, Göttingen, Germany) with 4x objective (Zeiss, NA: 0.01). First, background correction was applied in Fiji ImageJ software package (Calculator Plus) (build 1.53c Java version 1.8.0_172, NIH, USA), then QuPath measurement (version 0.3.0 Java version 16.0.2) was performed. Images were determined as brightfield photos and preprocessed to estimate stain vectors. Fast cell counts were performed using hematoxylin cell detection channel because of the similar color properties. The number of detections was represented in case of each image. To support the qualitative results by a quantitative method, Crystal Violet dye was solubilized from the cells with 1% SDS solution for 1 hour during gentle shaking. The optical density of the retrieved dye was measure at 570 nm in ClarioStar® *Plus* plate reader (BMG Labtech, Ortenberg, Germany) and value indicates the initial attached cell number. The workflow scheme is represented in **Supplemental Figure 9B**.

Apoptosis/Necrosis assay of mouse PSCs

Untreated and CM5480 treated PSCs were plated into 6 cm tissue culture dishes and maintained for 1 week then living cell/ apoptotic cell/ necrotic cell ratio was determined by Apoptosis/Necrosis Assay Kit. The cell surface was washed with 1x DPBS once, then cells were incubated for 5 min at 37°C incubator in TrypLE Express reagent. Cells were gently suspended and collected in a 15 ml centrifuge tube with 3 ml wash medium. Samples were washed with 1x DPBS two times with a centrifugation step (1000 rpm, 15°C, 5 min) between

each step. Cells were fixed with 4% paraformaldehyde for 10 min on 37°C. Pellet was washed in Assay buffer and incubated with Apoptosis-Necrosis antibody cocktail for 30 min in dark at RT (In Assay buffer (1000 µl) 10 µl Apopxin Green Indicator, 5 µl 7-AAD, 2 µl DAPI). After gently washing two times in Assay buffer, fluorescence intensity was measured in plate reader using a 96-well non-binding black microplate as follows: Necrosis: 7-AAD red (546→647nm); Apoptosis: Apopxin Green Indicator green (490→525 nm); Live cells: DAPI blue (359→461 nm).

Flow cytometry analysis of mouse PSCs

Untreated and CM5480 treated PSCs were plated into 6 cm tissue culture dishes and maintained for 1 week. The culture medium was removed from cells and replaced with Bromodeoxyuridine (BrdU) labelling solution (10 µM BrdU in PSC medium), then incubated for 1 hour in a 37°C humidified (5% CO_2) incubator. The cell surface was washed with 1x DPBS once, then cells were incubated for 5 min at 37°C incubator in TrypLE Express reagent. Cells were gently suspended and collected in a 15 ml centrifuge tube with 3 ml wash medium. Samples were washed with 1x DPBS two times with a centrifugation step (1000 rpm, 15°C, 5 min) between each step and the pellet was loosened gently. 100 µl 1x DPBS was added to each sample. While vortexing, 900 µl ice-cold 70% Ethanol was added dropwise and incubated on ice for 20 min. Cells were washed with 5 ml wash buffer (1x DPBS containing 0.5% BSA) and centrifuged at 1000 rpm for 5 min (RT). The supernatant was aspirated and the pellet was resuspended in denaturation solution (2M HCl), mixed well and incubated for 20 min at RT. Cells were washed with 5 ml wash buffer and centrifuged at 1000 rpm for 5 min (RT). The supernatant was aspirated and the pellet was resuspended in 0.5 ml 0.1M Sodium Borate (pH 8.5) to neutralize any residual acid, incubated for 2 min at RT. Cells were washed with 5 ml wash buffer and centrifuged at 1000 rpm for 5 min (RT). The supernatant was aspirated and anti-BrdU primary

antibody was added (anti-BrdU monoclonal antibody, Invitrogen, MA3-071). Cells were incubated for 1 hour at RT. Every 5-10 min cell pellet was moved as the cells settled to the bottom of the tube. Cells were washed with 5 ml wash buffer and centrifuged at 1000 rpm for 5 min (RT). The supernatant was aspirated, the secondary antibody was added (Donkey Anti-Mouse Alexa Fluor 647, Invitrogen, A31571) and incubated 45 min at RT in dark. Every 5-10 min cell pellet was moved as the cells settled to the bottom of the tube. Cells were washed with 5 ml wash buffer and centrifuged at 1000 rpm for 5 min (RT). To label the DNA content of nuclei, DAPI was applied in 1 µg/ml final concentration and incubated for a minimum of 30 min at RT in dark. Cells were filtered through a 70 µm pore size filter and transferred into 5 ml round bottom test tubes with 1x DPBS. Samples were loaded into BD FACSAria Fusion Flow Cytometer (BD Biosciences, CA, USA) and data were analyzed by BD FACSDiva Software (RRID:SCR_001456). During measurements, 20,000 events (cells) from each sample were detected and the obtained values were analyzed as follows. Cell populations were selected and grouped based on the cell size and DNA content and these designated points are plotted on a dot plot where the cell populations in different cell cycle phases are separated (P1: S phase; P2: G2/M phase; P3: G0/G1 phase). The data measured in this way were then compiled to give the percentage of cells in the different phases as follows: P1 or P2 or P3 / total * 100. AF647-A (anti-BrdU antibody + Alexa Fluor 647 antibody intensity) and DAPI-A (DAPI intensity) indicate the channels in which signals in each wavelength range are detected. FSC-A intensity indicates the diameters of the cells and was used for the discrimination of cells by size, while SSC-A provided information about the internal complexity of the cells.

Image analysis

Immunohistochemistry. Immunohistochemical image analysis was performed by QuPath software (version 0.3.0 Java version 16.0.2). First, background correction was applied in Fiji

ImageJ software package (Calculator Plus) (build 1.53c Java version 1.8.0_172, NIH, USA). All images were preprocessed by stain vector estimation then positive cell detection was applied with optimized and standardized values in case of each staining by the different antibodies. The proportion of positive cells (Positive (%)) represent the number of cells staining positive for the antibody of interest divided by the whole cell number (ie. hematoxylin positive cells). In case of SARAF staining, positive signals were separated by the IHC toolbox plugin (8) (Fiji ImageJ software, build 1.53c Java version 1.8.0_172, NIH, USA) as DAB (brown) signals and then measured by QuPath software (version 0.3.0 Java version 16.0.2). SARAF graphs represent the number of detected positive signals. To visualize better the positive signals in case of SARAF, alpha-Smooth Muscle Actin and GFAP staining, the color of the images was reversed by PhotoScape X software (version 4.2.1) increasing the contrast and applying a negative filter. This color modification lightest areas of the image appear darkest and the darkest areas appear lightest to help see the positive signals. SARAF protein sub-acinar localization was also visualized by PhotoScape X software (version 4.2.1). The original image was transformed to grayscale, while DAB positive signals were separated from the original image as well and re-colored as magenta. The grayscale and magenta images were merged.

Crossmon's Trichrome staining. Illustrative images of Crossmon's staining were captured by Zeiss Axio Imager.M2 microscope (Carl Zeiss Microscopy, Göttingen, Germany) with 2.5X objective (Zeiss, NA: 0.06). For automated evaluation of fibrosis, 5 random fields per Crossmon's Trichrome stained slides were captured at 20x magnification (Zeiss, NA: 0.8); large vascular areas and tissue edges were avoided to increase specificity of analysis. Slides from a minimum of 10 mice per experimental groups were analyzed. First, manual background correction was applied on every image using the Calculator Plus plugin in Fiji ImageJ software package (build 1.53c Java version 1.8.0_172, NIH, USA) as previously described (9). Then automated color deconvolution and image analysis was performed using a customized KNIME

(KNIME AG, Switzerland) workflow, utilizing the KNIME Image Processing - ImageJ Extension and the ColorDeconvolutionIJ2FromValues node. To determine the optimal vectors for Crossmon's Trichrome staining, we combined two images of slides stained only with either anilin blue or fuchsin dyes, applied a Gaussian blur filter to get an even color intensity and run a manual color deconvolution in ImageJ based on ROIs of the most representatively stained areas. The determined vectors were used for automated color deconvolution in the KNIME extension, where "color 1" represented the cytoplasm, "color 2" the collagen fibers. Threshold values of 215 and 105 were set for "color 1" and "color 2" the 32bit grayscale images, respectively. After thresholding, the sum of positive and negative pixels was counted and relative percentages were calculated; negative pixels of "color 1" images correlate with the area of total tissue (stained cytoplasm), the positive pixels of the same image correlate with the edema and/or adipose tissue (unstained area), while negative pixels of "color2" images correlate with the amount of fibrosis (stained collagen fibers).

Immunofluorescence staining. Images were modified to RGB Color images. Red-Green-Blue (RGB) color intensity was measured by RGB Measure plugin in Fiji ImageJ software package (build 1.53c Java version 1.8.0_172, NIH, USA) and values were indicated as Integrated Density % proportionate to each other. Sub-acinar and sub-ductal localization of proteins were determined by Plot profile analysis in Fiji, visualizing the detected gray values with respect to the current distance in GraphPad software as XY graph type (connecting line only).

PARP and Phospho-Histone H3 (Ser10) staining in mouse pancreatic tissue. Images were analyzed in Fiji ImageJ software (build 1.53c Java version 1.8.0_172, NIH, USA). The number of DAPI-positive cell nuclei and Phospho-Histone H3 (Ser10) (pHH3) -positive cells were counted manually, using Cell Counter plugin. DAPI/pHH3 ratios were represented in case of minimum 4 images per biological sample. Photos were taken from different areas of pancreatic

tissue that showed similar cell density. Areas were chosen only by DAPI, entire analysis was carried out blind in terms of pHH3.

Orai1 foci analysis on control and CM5480 treated mouse PSCs. 3D Foci Picker plugin by Fiji ImageJ software package (build 1.53c Java version 1.8.0_172, NIH, USA) was used to identify the number of puncta/μm^2 and intensity values per cell. The tolerance setting and Minimum pixels number in the focus were the same in case of each analyzed cell. The area of each single cell was measured and the number of Orai1 puncta was normalized to this value. A minimum of ten cells from the control and CM5480 treated group was counted and analyzed.

Explanatory images were created with BioRender.com.

Supplemental references

1. Maléth J, et al. Alcohol Disrupts Levels and Function of the Cystic Fibrosis Transmembrane Conductance Regulator to Promote Development of Pancreatitis. *Gastroenterology* 2015;148(2):427-439.e16.

2. Fanczal J, et al. TRPM2-mediated extracellular Ca2+ entry promotes acinar cell necrosis in biliary acute pancreatitis. *J. Physiol.* 2020;598(6):1253–1270.

3. Pallagi P, et al. Bile acid- and ethanol-mediated activation of Orai1 damages pancreatic ductal secretion in acute pancreatitis. *J. Physiol.* 2022;600(7):1631–1650.

4. Madácsy T, et al. Impaired regulation of PMCA activity by defective CFTR expression promotes epithelial cell damage in alcoholic pancreatitis and hepatitis. *Cell. Mol. Life Sci.* 2022;79(5):265.

5. Molnár R, et al. Mouse pancreatic ductal organoid culture as a relevant model to study exocrine pancreatic ion secretion. *Lab. Invest.* 2020;100(1):84–97.

6. Liang C-C, Park AY, Guan J-L. In vitro scratch assay: a convenient and inexpensive

method for analysis of cell migration in vitro. *Nat. Protoc.* 2007;2(2):329–333.

7. Feoktistova M, Geserick P, Leverkus M. Crystal Violet Assay for Determining Viability of Cultured Cells. *Cold Spring Harb. Protoc.* 2016;2016(4):pdb.prot087379.

8. Shu J, et al. Biomarker Detection in Whole Slide Imaging based on Statistical Color Models. *MIDAS J.* [published online ahead of print: September 3, 2010]; doi:10.54294/ix17bt

9. Landini G. How to correct background illumination in brightfield microscopy.2006;https://imagejdocu.list.lu/howto/working/how_to_correct_background_illumination_in_brightfield_microscopy. cited

Supplemental figures and figure legends

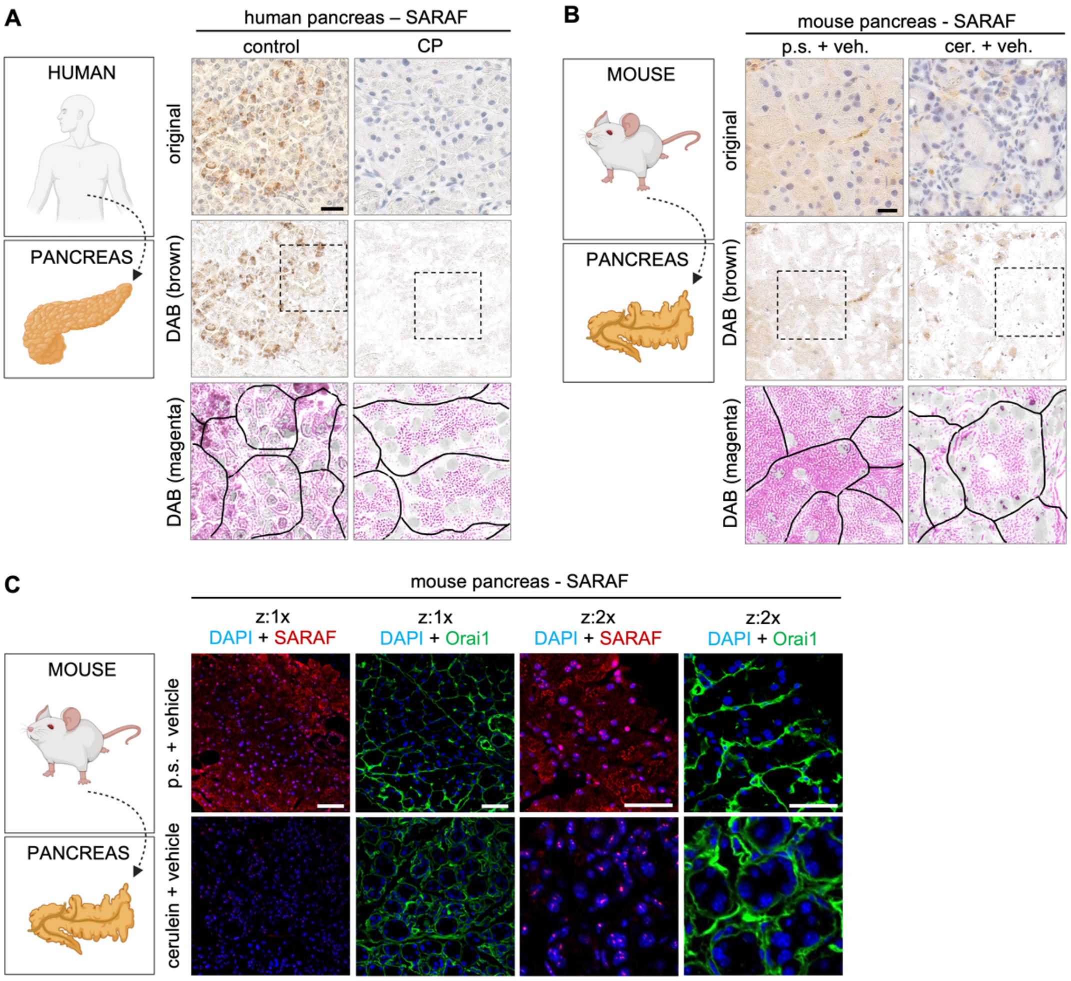

Supplemental Figure 1. Cerulein-treated mice showed decreased SARAF protein expression. (**A**) Representative IHC and inverted images of SARAF in the human pancreas. Positive signals of SARAF protein can be seen as DAB (brown) and magenta (for better visualization). Dashed line rectangles indicate the close-up regions on each image. Black traces mark the edge of acini. Scale bar: 20 µm (*n=3*/group, 5-7 images/human). (**B**) Representative IHC and inverted images of SARAF in the pancreas of control and CP mice (5x8 cerulein injections; 50 µg/bwkg). Positive signals of SARAF protein can be seen as DAB (brown) and magenta (for better visualization) (*n=3*/group, 5-7 images/animal). Dashed line rectangles indicate the close-up regions on each image. Black traces mark the edge of acini. Scale bar: 20 µm. (**C**) Immunofluorescence staining of SARAF+DAPI and Orai1+DAPI in control (p.s. + vehicle) and cerulein-treated (cerulein + vehicle) mouse pancreas (*n=3*/group, 5-7 images/animal). Scale bars: 50 µm. Explanatory images were created with BioRender. Abbreviations: cer.: cerulein; CP: chronic pancreatitis; p.s.: physiological saline; veh.: vehicle.

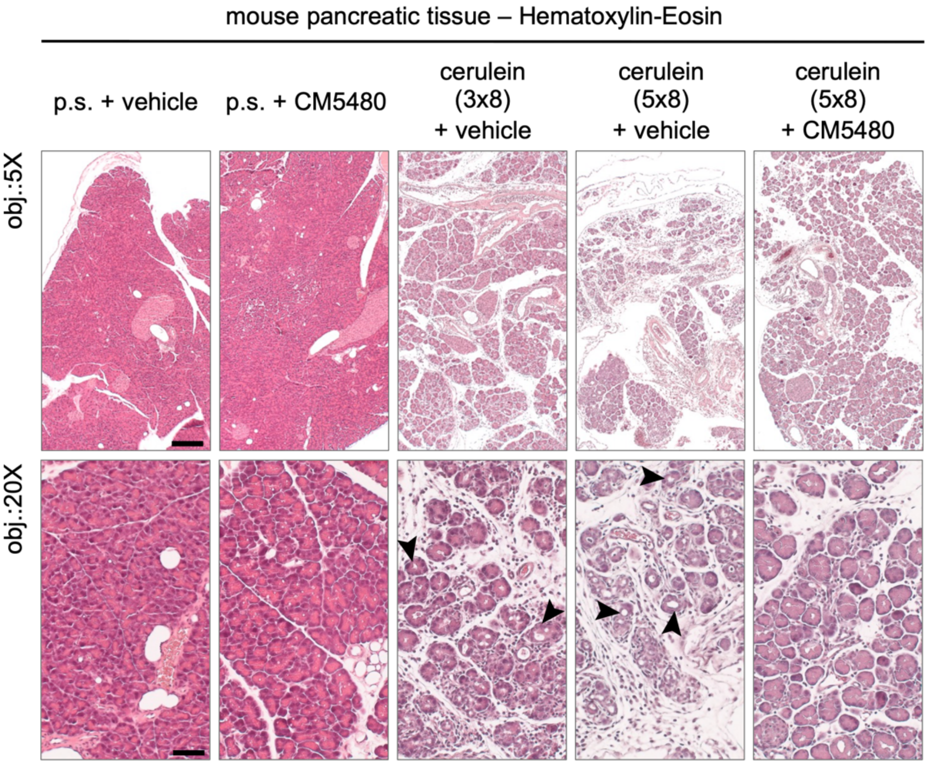

Supplemental Figure 2. Cerulein-treated mice showed increased pancreatic fibrosis. Representative images of Hematoxylin-Eosin staining of mouse pancreatic tissues. CP was induced by 3x8 and 5x8 cerulein (50 µg/bwkg), CM5480 was administrated on the last 5 consecutive days of the experiment (*n=6-10*/group, 5-7 images/animal). Scale bar: 200 µm (objective: 5x) and 50 µm (objective: 20x), respectively. Black arrowheads indicate the pancreatic acinar cells that underwent acinar-to-ductal metaplasia. Abbreviations: obj.: objective; p.s.: physiological saline.

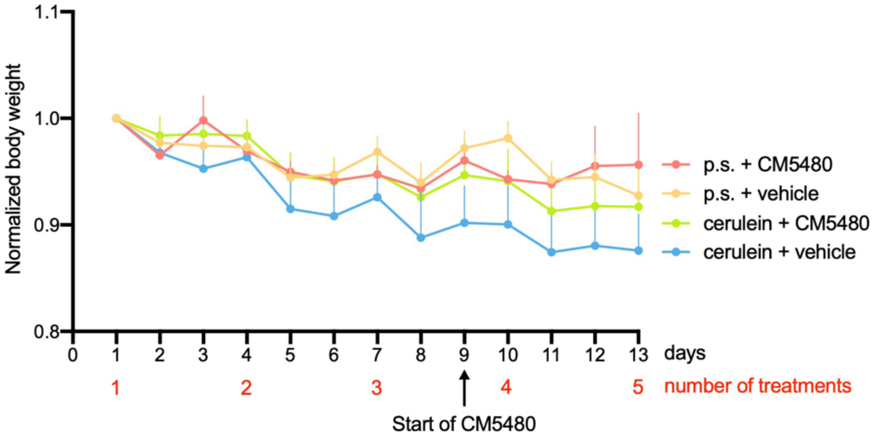

Supplemental Figure 3. Orai1 inhibition reduced pancreas weight/body weight ratio in cerulein-treated mice. Curves shows the changes in body weight during the course of cerulein-based CP induction (red numbers) and CM5480 treatment (*n=6-10*/group, 5-7 images/animal). Abbreviations: p.s.: physiological saline.

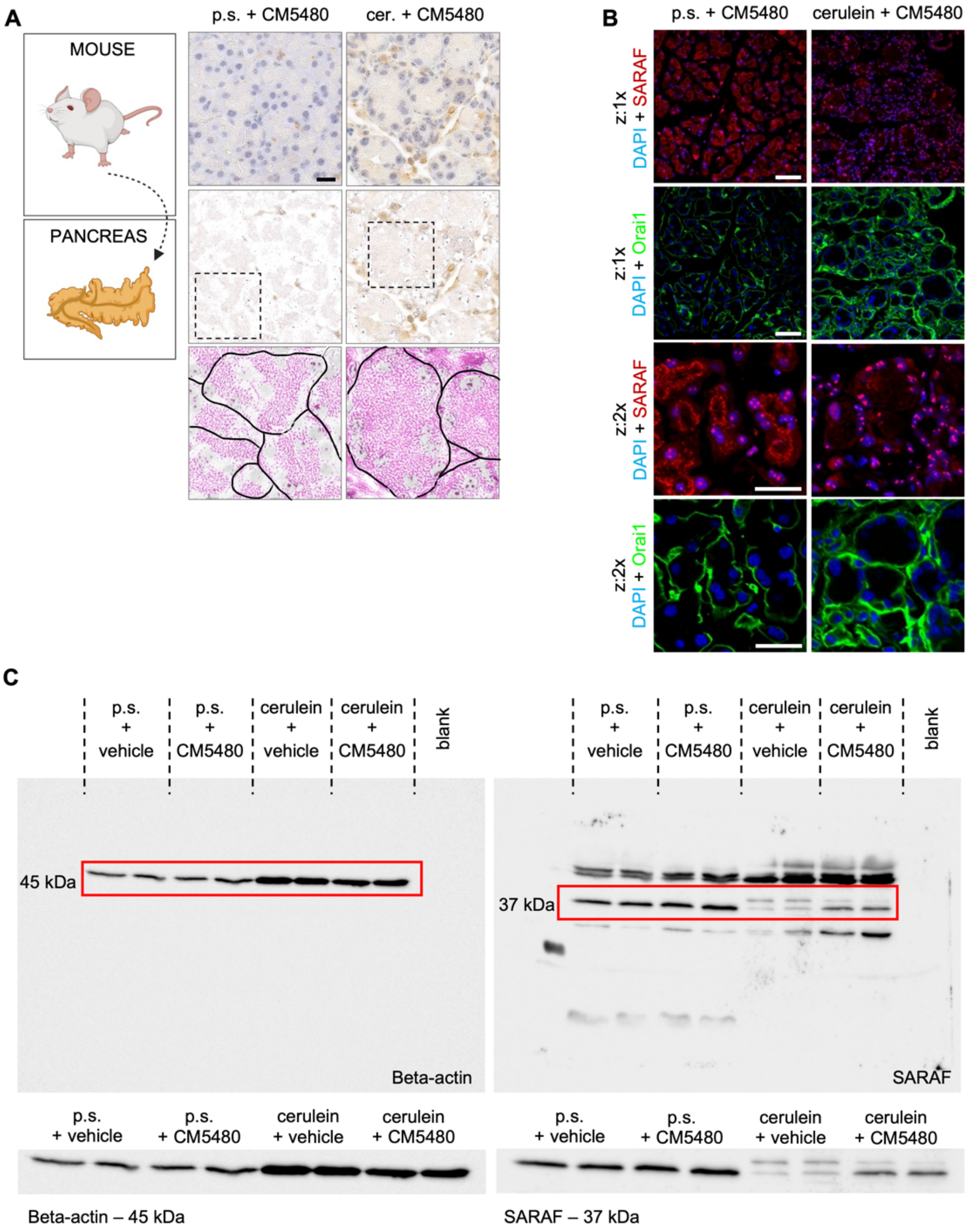

Supplemental Figure 4. SARAF protein expression was decreased in cerulein-treated mice but partially restored due to Orai1 inhibition. (**A**) Representative IHC and inverted images of SARAF in the pancreas of control + CM5480 (p.s. + CM5480) and CP + CM5480 (cer. + CM5480) mice (5x8 cerulein injections; 50 µg/bwkg; 5-days post-treatment with CM5480 20 mg/bwkg). Positive signals of SARAF protein can be seen as DAB (brown) and magenta (for better visualization) (*n=3*/group, 5-7 images/animal). Dashed line rectangles indicate the close-up regions on each image. Black traces mark the edge of acini. Scale bar: 20 µm. (**B**) Immunofluorescence staining of SARAF + DAPI and Orai1 + DAPI in control + CM5480 (p.s. + CM5480) and CP + CM5480 (cer. + CM5480) mouse pancreas (*n=3*/group, 5-7 images/animal). (**C**) Full unedited blots for Figure 4 showing the western blot analysis of SARAF and beta-actin protein level: protein samples were isolated from mouse pancreas in each group and loaded in 20 µg (*n=6*/group). Red frames indicate which bands were used for the Figure 4. Scale bars: 50 µm. Abbreviations: p.s.: physiological saline; z: zoom.

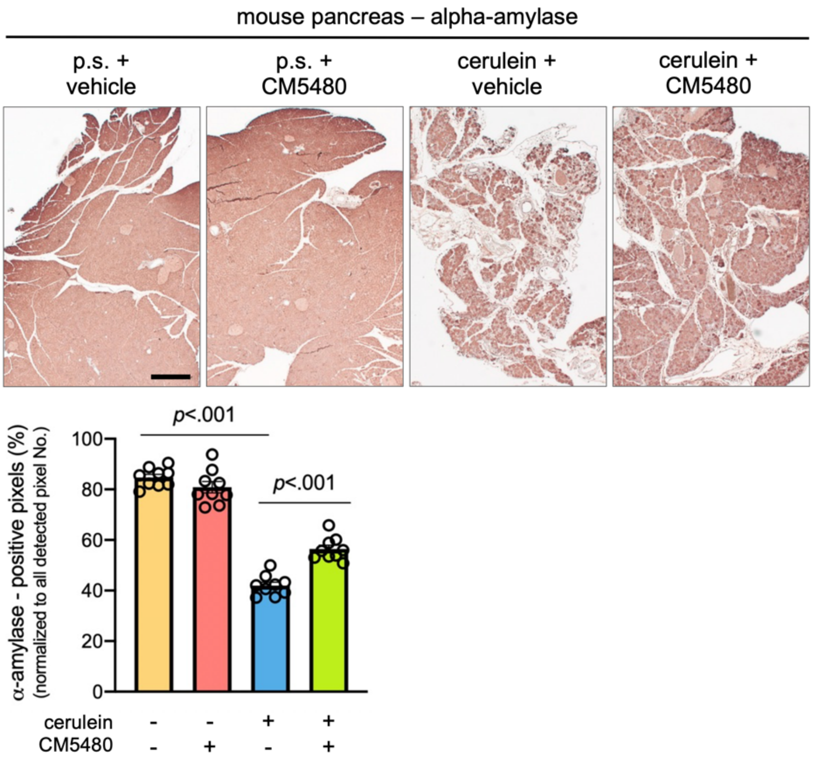

Supplemental Figure 5. CM5480 treatment preserved the alpha-amylase expression and acini structure during chronic pancreatitis. Representative IHC images of pancreatic alpha-amylase in mouse pancreatic tissues (*n=3*/group, 5-7 images/animal). Scale bar: 500 µm. The bar graph shows the detected positive pixels indicating the presence of alpha-amylase in pancreatic tissue. A P value less than 0.05 was considered significant by ordinary one-way ANOVA. Data represent mean ± SEM. Abbreviations: p.s.: physiological saline.

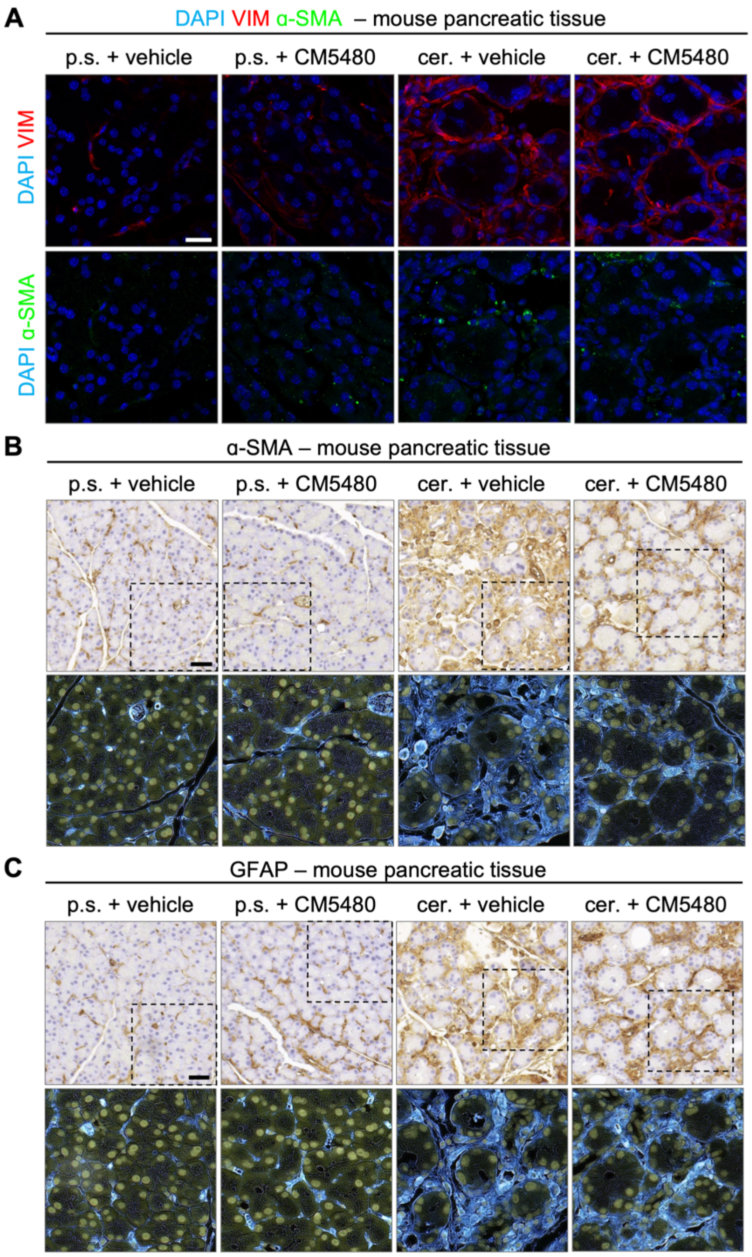

Supplemental Figure 6. Stellate cell activation was inhibited by CM5480. (**A**) Immunofluorescence staining of alpha-smooth muscle actin (α-SMA) and vimentin (VIM) in mouse pancreatic tissue (*n=3*/group, 5-7 images/animal). Scale bar: 20 µm. (**B**) IHC images of α-SMA in mouse pancreatic tissues (*n=3*/group, 5-7 images/animal). Scale bar: 50 µm. Area in the dashed square were inverted to visualize the presence of α-SMA and GFAP protein around acini. (**C**) IHC images of glial fibrillary acidic protein (GFAP) in mouse pancreatic tissues (*n=3*/group, 5-7 images/animal). Scale bar: 50 µm. Area in the dashed square were inverted to visualize the presence of α-SMA and GFAP protein around acini. Abbreviations: cer.: cerulein; p.s.: physiological saline.

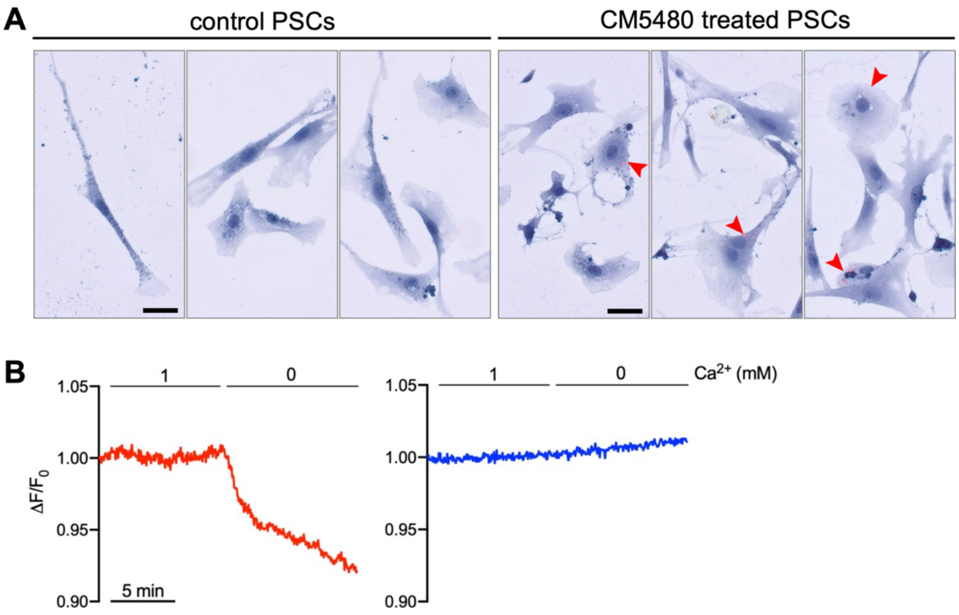

Supplemental Figure 7. The amount of lipid-droplet-containing stellate cells increased due to CM5480 treatment. (**A**) Representative images of Oil Red O positive and negative cells in cultured PSCs (*n=4*/group from 4 animals, 5-7 images/culture). Scale bar: 20 µm. (**B**) Representative Ca^{2+} traces of control fibroblast cultures (*n=4* cultures from 4 animals). Abbreviations: PSC: pancreatic stellate cells.

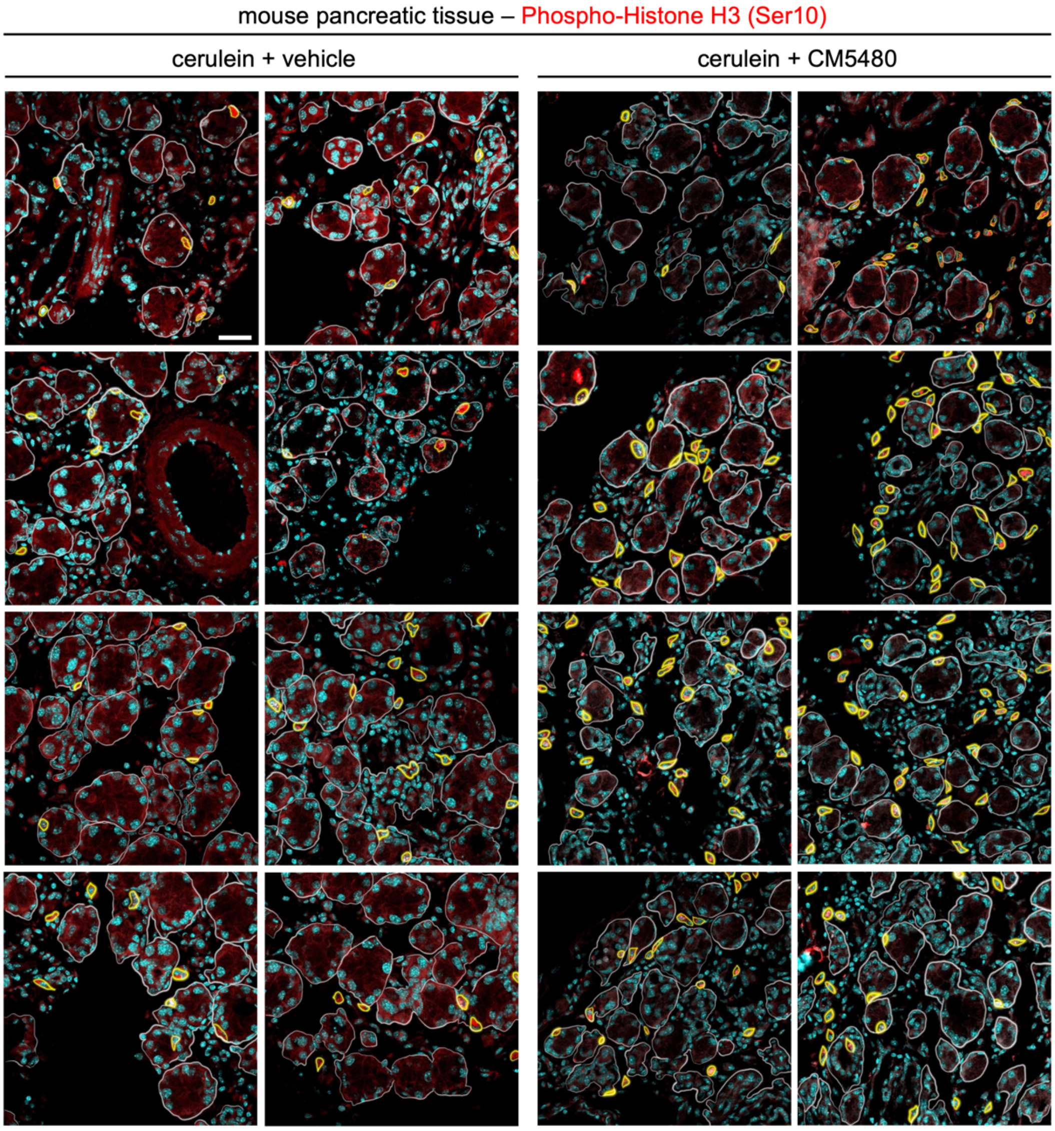

Supplemental Figure 8. CM5480 increases the number of Phospho-Histone H3 positive cells in vivo. Immunofluorescence staining of Phospho-Histone H3 (Ser10) (pHH3) in mouse pancreatic tissue (*n=3*/group, 5-7 images/animal). Red color shows the pHH3 staining; cyan color indicates the nuclei counterstained with DAPI; white lines show the edges of well-defined structures, like acini; yellow lines surround the pHH3-positive cells. Scale bar: 50 µm.

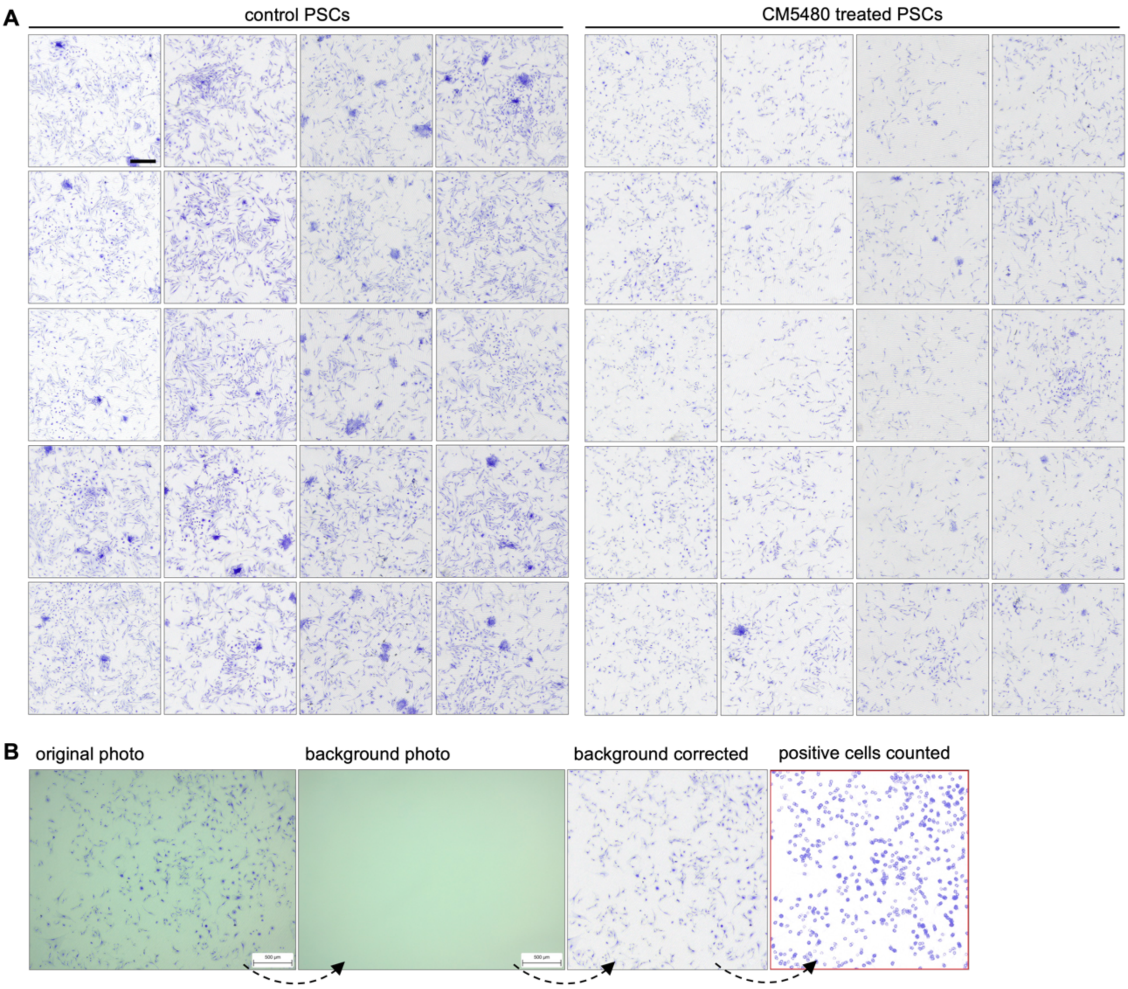

Supplemental Figure 9. Due to Orai1 inhibition, the number of cells in PSC cultures was reduced. (**A**) Crystal Violet staining of control and CM5480 treated PSCs on day 7 of culturing. Shown images are randomly selected areas of the PSC cultures isolated from 4 animals (*n=5*/group from 4 animals). Scale bar: 500 µm. (**B**) Steps of the image analysis that was used for the Crystal Violet assay evaluation.

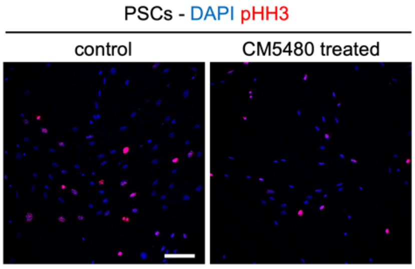

Supplemental Figure 10. The number of Phospho-Histone H3 (Ser10) positive cells did not change in in vitro stellate cell cultures due to Orai1 inhibition. Immunofluorescence staining of Phospho-Histone H3 (Ser10) **(**pHH3) and nuclei (DAPI) in control and CM5480 treated PSCs (*n=5*/group from 4 animals). Scale bar: 50 µm. Abbreviations: PSC: pancreatic stellate cells.

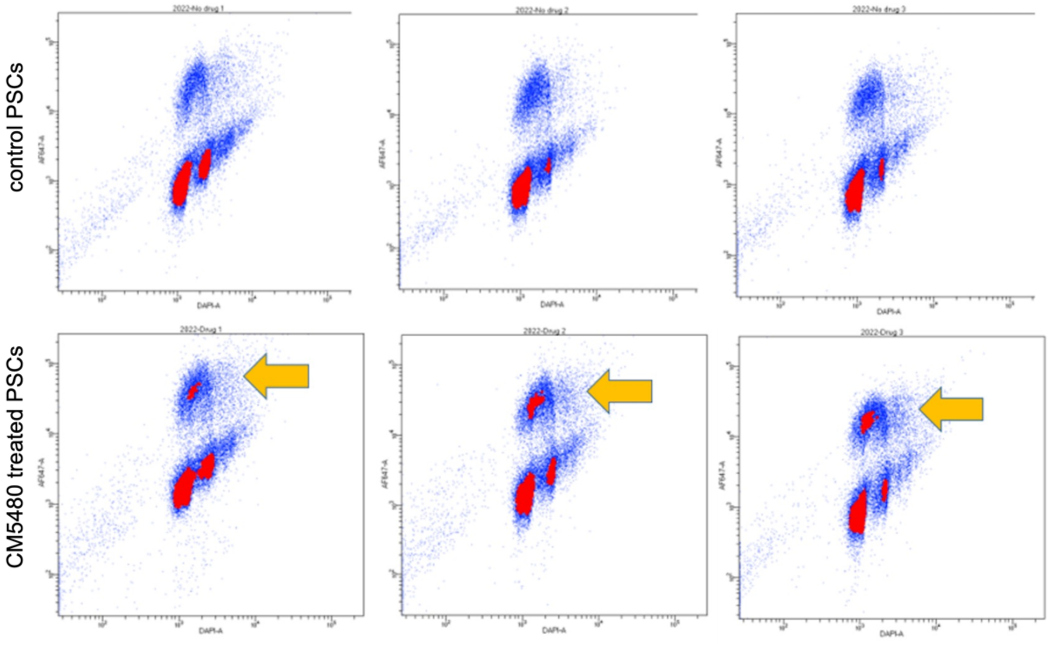

Supplemental Figure 11. PSC proliferation was impaired by Orai1 inhibition, which caused a cell cycle arrest and accumulation in the S phase. Flow cytometry was used to examine cell proliferation in control and CM5480-treated PSCs using BrdU (Alexa Fluor 647) and DAPI (DNA content) staining. Cell population accumulated in S-phase is indicated by yellow arrows in CM5480-treated culture replicates (*n=3*/group from 3 animals). Abbreviations: PSC: pancreatic stellate cells.

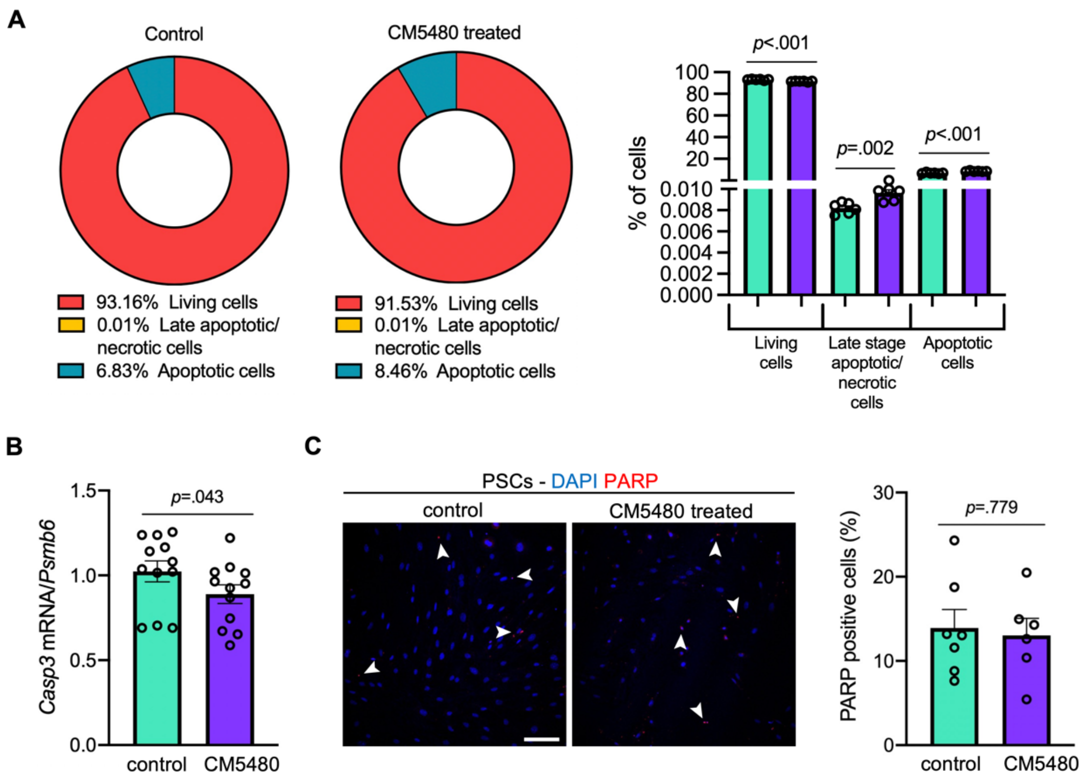

Supplemental Figure 12. The level of apoptosis did not change upon CM5480 treatment. (**A**) Apoptosis/necrosis/Living Cells assay of control and CM5480 treated PSCs at day 7 of culturing. Parts of whole diagram and bar graph show the percentage data of apoptosis/late-apoptosis OR necrosis/living cells (*n=4*/group from 4 animals). (**B**) mRNA expression of *Casp3* in cultured PSCs (*n=4*/group from 4 animals). (**C**) Immunofluorescence staining of PARP in mouse PSC cultures. White arrowheads indicate the PARP-positive cells. The bar graph indicates the number of PARP-positive cells compared to the whole cell number. (*n=5*/group from 4 animals, 5-7 images/culture). Scale bar: 50 µm. A P value less than 0.05 was considered significant by unpaired t test (**A**, **C**) and Mann-Whitney test (**B**). Data represent mean ± SEM. Abbreviations: PSC: pancreatic stellate cells.

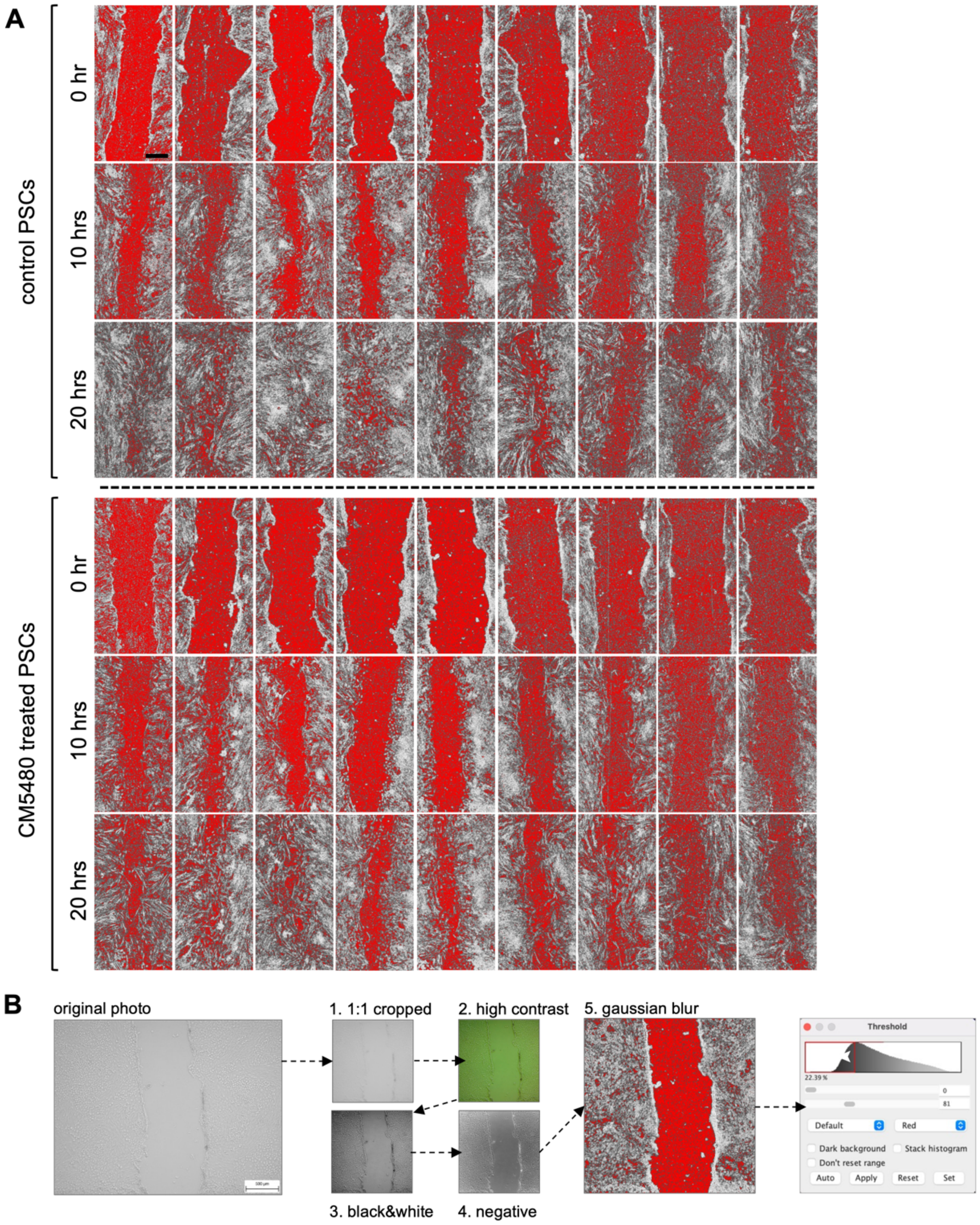

Supplemental Figure 13. CM5480 decelerated the speed of migration in PSC cultures. (**A**) Wound healing of cultured PSCs. Representative images and graph show the percentage of wound closure after 0, 10 and 20 hours (*n=2*/group from 9 animals). Scale bar: 500 µm. (**B**) Shown is the workflow that was used for the wound healing assay evaluation. Abbreviations: PSC: pancreatic stellate cells.

Supplemental Table 1 – Materials used during experiments listed (A-D)

Name	Provider	Cat. No.
10x TG Buffer	Bio-Rad	161-0771
10X TGS	Bio-Rad	161-0772
16% Formaldehyde	Thermo Scientific	28906
16% Paraformaldehyde	Alfa Aesar	11400580
2-Mercaptoethanol	Sigma-Aldrich	M6250-100ML
30% Acrylamide/Bis Solution 29:1	Bio-Rad	1610156
5-Bromo-2 Deoxyuridine	Invitrogen	B23151
96% Ethanol	Molar Chemicals	02911-481-410
Acetic acid	Sigma-Aldrich	A6283-1L
Alpha-Amylase Assay kit	Diagnosticum	47462
Alum hematoxylin	Reanal	08004-6-25
Ammonium Persulphate	Thermo Scientific	17874
Anilin blue	Thomasker	13381-25G
Apoptosis/Necrosis Assay kit (blue/green/red)	Abcam	ab176749
BCECF,AM	Invitrogen	B1170
Bond™ Dewax Solution	Leica Biosystems	AR9222
Bond™ Epitope Retrieval	Leica Biosystems	AR9640
Bond™ Polymer Refine Detection	Leica Biosystems	DS9800
Bond™ Primary Antibody Diluent	Leica Biosystems	AR9352
Bond™ Wash Solution	Leica Biosystems	AR9590
Boric acid	Sigma-Aldrich	B6768-1KG
Bovine Serum Albumin	Pan-Biotech	P06-1391500
Bromophenol Blue	Sigma-Aldrich	B0126-25G
Caerulein ammonium salt	Bachem AG	4030451.0005
Clarity Western ECL Substrate	Bio-Rad	170-5060
CM5480	Provided by CalciMedica	
Collagenase	Worthington	LS005273
Collagenase IV	Gibco	17104019
cOmplete ULTRA protease inh. cocktail	Roche	05892970001
Coomassie® Brilliant Blue	Merck	1.15444.0025
Corn cob bedding - REHOFIX	JRS	REHOFIX MK2000
CPA	Tocris	1235
Crystal Violet	Sigma-Aldrich	C0775
DAPI	Sigma-Alrich	MBD0015-5ML
DMEM/F12	Sigma-Aldrich	D6421
DPBS (1X)	Gibco	14190-094

Supplemental Table 2 – Materials used during experiments listed (E-R)

Name	Provider	Cat.No.
EnzCheck™ Elastase Assay kit	Invitrogen	E12056
Eosin Y Alcoholic Solution	Leica Biosystems	3801600E
Eukitt® Quick-hardening mounting medium	Sigma-Aldrich	03989-100ML
Fluoromount™ Aqueous Mounting Medium	Sigma-Aldrich	F4680
Fuchsin acid	Gyógyszeralapanyag Készletező V.	2-06-073-4-15
FURA-2,AM	Invitrogen	F1201
Glycerol	Sigma-Aldrich	G5516-1L
Hank's Balanced Salt Solution (HBSS)	Sigma-Aldrich	H9269
Harrys Hematoxylin	Leica Biosystems	3801560E
HEPES	Sigma-Aldrich	H3375
Hydrochloric Acid (HCl)	Sigma-Aldrich	258148
Hydroxyproline Assay kit	Sigma-Aldrich	MAK008
iScript™ cDNA Synthesis Kit	Bio-Rad	178890
Isopropyl alcohol	Sigma-Aldrich	19516-500ML
Ketamine	-	-
Media 199	Gibco	11150059
Methanol	Sigma-Aldrich	34860
MQAE	Invitrogen	E3101
Novocastra Peroxidase Block	Leica Biosystems	RE7101-CE
NucleoSpin RNA Plus kit	Macherey-Nagel	740955.250
NucleoSpin totalRNA FFPE XS Kit	Macherey-Nagel	740969
NucleoZOL	Macherey-Nagel	740404.200
Oil Red O Stain Kit	Abcam	ab150678
Orange G	Reanal	16230
Pentobarbital	-	-
Phosphoric Acid	Sigma-Aldrich	30416-1L-M
Phosphotungstic acid	Reanal	06101
PhosSTOP phosphatase inhibitor	Roche	04906845001
Picric acid	Sigma-Aldrich	197378
Pierce BCA Protein Assay Kit	Thermo Scientific	23225
Pluronic™ F-127	Invitrogen	P3000MP
Poly-L-lysine	Sigma-Aldrich	P4707-50ML
Precision Plus Protein WesternC Standards	Bio-Rad	161-0376
RIPA Lysis Buffer	Merck Millipore	20-188
RNAlater™	Sigma-Aldrich	R0901-100ML

Supplemental Table 3 – Materials used during experiments listed (S-X)

Name	Provider	Cat.No.
Secretin	Sigma-Aldrich	S7147
Shandon CryoMatrix	Thermo Scientific	6769006
Sodium Chloride	Sigma-Aldrich	S9888-2.5KG
Sodium Citrate	Sigma-Aldrich	C8532
Sodium Dodecyl Sulfate	Sigma-Aldrich	L4509
Soybean Trypsin Inhibitor	Gibco	17075029
SsoAdvanced™ Universal SYBR® Green Supermix	Bio-Rad	172-5271
Sucrose	Sigma-Aldrich	S0389-500G
Triton X100	Reanal	32190-1-99-33
TrypLE™ Express	Gibco	12605028
Tween20	Sigma-Aldrich	P1379
UltraPure TEMED	Invitrogen	15524010
VRF1(P) standard rodent food	Special Diets Services	801900
Xylazine	-	-

Supplemental Table 4 – Tools used during experiments listed

Name	Provider	Cat.No.
0.22 µm filter	Labex	FBS30PES022S
5 mL Polystyrene Round-Bottom Tube	Falcon - Corning	352054
Cell Culture Dish, PS, 100/20 mm	Greiner Bio-One	664160
Cell Culture Multiwell plate 12 well	Greiner Bio-One	665180
Cell Culture Multiwell plate, 6 well	Greiner Bio-One	657160
Cell Culture Dish, PS, 60/15 mm	Greiner Bio-One	628160
Cover slip (24x24 mm)	VWR	631-1583
Cover slip (24X50 mm)	VWR	631-0146
Easypet® 3 – Electronic Pipette Controller	Eppendorf	4430000018
EASYstrainer 100 µM	Greiner Bio-One	542000
EASYstrainer 70 µm	Greiner Bio-One	542070
Eppendorf Safe-Lock Tubes	Eppendorf	30121589
Epredia™ SuperFrost Plus™ Adhesion slides	Thermo Scientific	J1800AMNZ
Finnpipette F1 Fixed Volume 10 ml	Thermo Scientific	4651120N
Microplate, 96 well, PS, F-bottom, black, non-binding	Greiner Bio-One	655900
Pipet-Lite™ XLS+ manual 8-channel pipette, 20-300 µL	Rainin	17013806
Pipetman L P10L, 1-10 µL	Gilson	FA10002M
Pipetman L P20L, 2-20 µL	Gilson	FA10003M
Pipetman L P100L, 10-100 µL	Gilson	FA10004M
Pipetman L P200L, 20-200 µL	Gilson	FA10005M
Pipetman L P1000L, 100-1000 µL	Gilson	FA10006M
Pipette Tips RC LTS, 300 µl	Rainin	17001132
PVDF Transfer Membrane	Thermo Scientific	88520
Reaction tube, 1.5 ml	Greiner Bio-One	616201
Sapphire Filter Tip, 10 µl	Greiner Bio-One	771353
Sapphire Filter Tip, 20 µl	Greiner Bio-One	773353
Sapphire Microplate, 96 well for RT PCR	Greiner Bio-One	669285
Sapphire PCR tube, 0.2 ml	Greiner Bio-One	683201
Serological pipette, 5 ml	Greiner Bio-One	606180
Serological pipette, 10 ml	Greiner Bio-One	607180
Serological pipette, 25 ml	Greiner Bio-One	760180
Sterican 18Gx2" nails	Braun	4667123
Syringe 20 ml	BD	300296
Tube, 50 ml, PP 30/115 mm	Greiner Bio-One	210261
Tube, 15 ml, PP 17/120 mm	Greiner Bio-One	188271
Universal Filter Tips 200-1000 µl	Greiner Bio-One	740288
Universal Filter Tips 20-200 µl	Greiner Bio-One	739288

Supplemental Table 5 – Splitting media of PSCs

Component	Manufacturer/Cat.No.	Final cc/volume
Advanced DMEM/F-12	Gibco, Cat.No. 12634-010	500 ml
1 M HEPES	Gibco, Cat.No. 15630080	10 mM (5 ml)
GlutaMAX Supplement (100X)	Gibco, Cat.No. 35050061	1 v/v%
Primocin (400X)	Invivogen, Cat.No. ant-pm-2	1.25 ml

Supplemental Table 6 – Digestion media of PSCs

Component	Manufacturer/Cat.No.	Final cc/volume
Splitting media	-	20 ml
Collagenase IV	Gibco, Cat.No. 17104019	1250 U/ml
Dispase II	Sigma-Aldrich, Cat.No. D4693-1G	0.5 U/ml
Fetal Bovine Serum	Gibco, Cat.No. 10500064	2.5 v/v%
Soybean Trypsin Inhibitor	Gibco, Cat.No. 17075029	1 mg/ml

Supplemental Table 7 – Wash media of PSCs

Component	Manufacturer/Cat.No.	Final cc/volume
Splitting media	-	20 ml
Fetal Bovine Serum	Gibco, Cat.No. 10500064	2.5 v/v%
Antibiotic-Antimycotic Solution (100X)	Gibco, Cat.No. 15240062	1 v/v%
Kanamycin Sulfate (100X)	Gibco, Cat.No. 15160047	1 v/v%
Voriconazole	Sigma-Aldrich, Cat.No. V-032-1ML	2 μg/ml

Supplemental Table 8 – Feeding media of PSCs

Component	Manufacturer/Cat.No.	Final cc/volume
Dulbecco′s Modified Eagle′s Medium/Nutrient Mixture F-12 Ham	Sigma-Aldrich, Cat.No. D6421	83 v/v%
Fetal Bovine Serum	Gibco, Cat.No. 10500064	15 v/v%
Antibiotic-Antimycotic Solution (100X)	Gibco, Cat.No. 15240062	1 v/v%
Kanamycin Sulfate (100X)	Gibco, Cat.No. 15160047	1 v/v%
Voriconazole	Sigma-Aldrich, Cat.No. V-032-1ML	2 µg/ml

Supplemental Table 9 - Composition of solutions used during fluorescent measurements

	Standard HEPES	Ca-Free HEPES	Standard HCO_3^-	NH_4Cl-HCO_3^-	Cl-Free HCO_3^-
**NaCl**	130	132	115	95	
**KCl**	5	5	5	5	
**$MgCl_2$**	1	1	1	1	
**$CaCl_2$**	1		1	1	
**Hepes**	10	10			
**Glucose**	10	10	10	10	10
**$NaHCO_3^-$**			25	25	25
**EGTA**		0.1			
**NH_4Cl**				20	
**Na-gluconate**					115
**K_2-sulphate**					2.5
**Ca-gluconate**					6
**Mg-gluconate**					1

Supplemental Table 10 – Primary antibodies used in IHC

Name	Source	Clonality	Cat.No.	Provider	Dilution
anti-SARAF (TMEM66)	Rabbit	Polyclonal	PA1-31588	Invitrogen	1:100
CD3	Mouse	Monoclonal	MA5-12577	Invitrogen	1:20
CD8	Rabbit	Polyclonal	PA588265	Invitrogen	1:100
CD19	Rabbit	Recombinant monoclonal	MA5-32560	Invitrogen	1:100
F4/80	Rabbit	monoclonal	70076	Cell Sign. Technology	1:200
Myeloperoxidase	Rabbit	Polyclonal	A0398	Dako	1:200
alpha-SMA	Mouse	Monoclonal	202M-94	Cell Marque	1:100
GFAP	Mouse	Monoclonal	BSB 5564	Bio SB	1:250
alpha-amylase	Rabbit	Polyclonal	ab199132	abcam	1:100

Supplemental Table 11 – Primary antibodies used during experiments

Name	Source	Clonality	Cat.No.	Provider
anti-alpha-SMA	Goat	Polyclonal	NB300-978	Novus Biologicals
anti-alpha-SMA	Mouse	Monoclonal	202M-94	Cell Marque
anti-beta-actin	Rabbit	Polyclonal	4967S	Cell Signal. Tech.
anti-BrdU	Mouse	Monoclonal	MA3-071	Invitrogen
anti-CFTR	Rabbit	Polyclonal	ACL-006	Alomone Labs
anti-GFAP	Chicken	Polyclonal	PA1-10004	Invitrogen
anti-GFAP	Mouse	Monoclonal	BSB 5564	Bio SB
anti-Occludin	Mouse	Monoclonal	33-1500	Invitrogen
anti-Orai1	Rabbit	Polyclonal	ab59330	Abcam
anti-Orai1	Mouse	Monoclonal	NBP1-75522	Novus Biologicals
anti-PARP	Mouse	Monoclonal	NB100-56599	Novus Biologicals
anti-pHH3	Rabbit	Polyclonal	06-570	Sigma-Aldrich
anti-TMEM66	Rabbit	Polyclonal	PA5-31588	Invitrogen
anti-Vimentin	Mouse	Monoclonal	MA3745	Invitrogen

Supplemental Table 12 – Secondary antibodies used during experiments

Name	Provider	Cat. No.
Donkey Anti-Goat IgG H&L (Alexa Fluor® 488)	abcam	ab150129
Donkey anti-Mouse IgG (H+L) Highly Cross-Adsorbed Secondary Antibody, Alexa Fluor 647	Invitrogen	A31571
F(ab')2-Goat anti-Mouse IgG (H+L) Cross-Adsorbed Secondary Antibody, Alexa Fluor Plus 488	Invitrogen	A48286
F(ab')2-Goat anti-Mouse IgG (H+L) Cross-Adsorbed Secondary Antibody, Alexa Fluor Plus 555	Invitrogen	A48287
F(ab')2-Goat anti-Mouse IgG (H+L) Cross-Adsorbed Secondary Antibody, Alexa Fluor Plus 647	Invitrogen	A48289
F(ab')2-Goat anti-Rabbit IgG (H+L) Cross-Adsorbed Secondary Antibody, Alexa Fluor Plus 488	Invitrogen	A48282
F(ab')2-Goat anti-Rabbit IgG (H+L) Cross-Adsorbed Secondary Antibody, Alexa Fluor Plus 647	Invitrogen	A48285
Goat anti-Chicken IgY (H+L) Cross-Adsorbed Secondary Antibody, Alexa Fluor Plus 647	Invitrogen	A32933
Goat anti-Rabbit IgG (H+L) Secondary Antibody, HRP conjugate	Invitrogen	31460

Supplemental Table 13 – Primary and Secondary antibody pairing during experiments

Primary ab.	Cat.No.	Dilution	Secondary ab.	Dilution
anti-beta-actin	4967S	1:10000	G-A-R HRP	1:10000
anti-BrdU	MA3-071	1:200	D-A-M 647	1:2000
anti-CFTR	ACL-006	1:200	G-A-R 488	1:2000
anti-GFAP	PA1-10004	1:200	G-A-Ch 647	1:2000
anti-Occludin	33-1500	1:200	G-A-M 647	1:2000
anti-Orai1	ab59330	1:100	G-A-R 488	1:2000
anti-Orai1	NBP1-75522	1:200	G-A-M 488	1:2000
anti-PARP	NB100-56599	1:200	G-A-M 647	1:2000
anti- pH3	06-570	1:200	G-A-R 647	1:2000
anti-TMEM66	PA5-31588	1:200	G-A-R 647	1:2000
anti-TMEM66	PA5-31588	1:1000	G-A-R HRP	1:10000
anti-alpha-SMA	NB300-978	1:200	D-A-G 488	1:1000
anti-Vimentin	MA3745	1:100	G-A-M 555/ D-A-M 647	1:2000

Supplemental Table 14 – Primers were used in qPCR (purchased from Bio Basic Canada Inc., Ontario, Canada)

Name	Forward (5'-3')	Reverse (5'-3')
Acta2	GGCTCTGGGCTCTGTAAGG	CTCTTGCTCTGGGCTTCATC
Casp3	AGCTTGGAACGGTACGCTAA	CCAGAGTCCACTGACTTGCT
Cftr	TAGGGGAAGTCACCAAGGCT	TGGGTGAAGAAGCAGTGTCC
Fn1	GTGGTGTAGCACAACTTCCA	AGTCTGAACCAAAACCGCAT
GAPDH	TCCAAAATCAAGTGGGGCGA	CAAATGAGCCCCAGCCTTCT
H3c4	CCGTTCTCATTCCCTGAGACT	GTAGCGGTGAGGCTTCTTCA
Il1b	GAGTGTGGATCCCAAGCAAT	ACGGATTCCATGGTGAAGTC
Ki-67	ATCATTGACCGCTCCTTTAGGT	GTATCTTGACCTTCCCCATCAGG
Orai1	CTTCGCCATGGTAGCGAT	TGTGGTGCAGGCACTAAAGA
Psmb6	CTGACAAGCTGACCCCTATC	TGGAAACCAAGCTGGTAAGT
Rpl13a	GAGGTCGGGTGGAAGTACCA	TGCATCTTGGCCTTTTCCTT
SARAF	GGCAGTGCTTTTACAGGACA	GGGTGTTGCCGCTCTATTG
Saraf	GGGTGATACAGTGCCAGAAC	TCGGTCTTACATTCCCACTGT
Tgfb1	ACGTCACTGGAGTTGTACGG	TTTGGGGCTGATCCCGTTG
Tnfa	CCCAAAGGGATGAGAAGTTCC	ACAGGCTTGTCACTCGAATTT

Supplemental Table 15 – Solutions and gel compositions used in western blot

15% Running gel		Stacking gel	
Name	Amount	Name	Amount
dH2O	2.2 ml	dH2O	2.975 ml
30%Bis-acrylamide	5 ml	30% Bis-acrylamide	0.67 ml
1.5M Tris pH 8.8	2.6 ml	0.5M Tris pH 6.8	1.25 ml
10% SDS	0.1 ml	10% SDS	0.05 ml
10% APS	0.1 ml	10% APS	0.05 ml
TEMED	0.01 ml	TEMED	0.005 ml

5x Laemmli buffer (total of 10 ml)	
Name	Amount
0.5M Tris pH 6.8	1.75 ml
Glycerol	4.5 ml
SDS (0.25 g dissolved in 1 ml dH2O)	2 ml 0.5 g total
0.25% Bromophenol blue (25 mg in 10 ml dH2O)	0.5 ml
2- Mercaptoethanol	1.25 ml

Full unedited blots for Figure 4

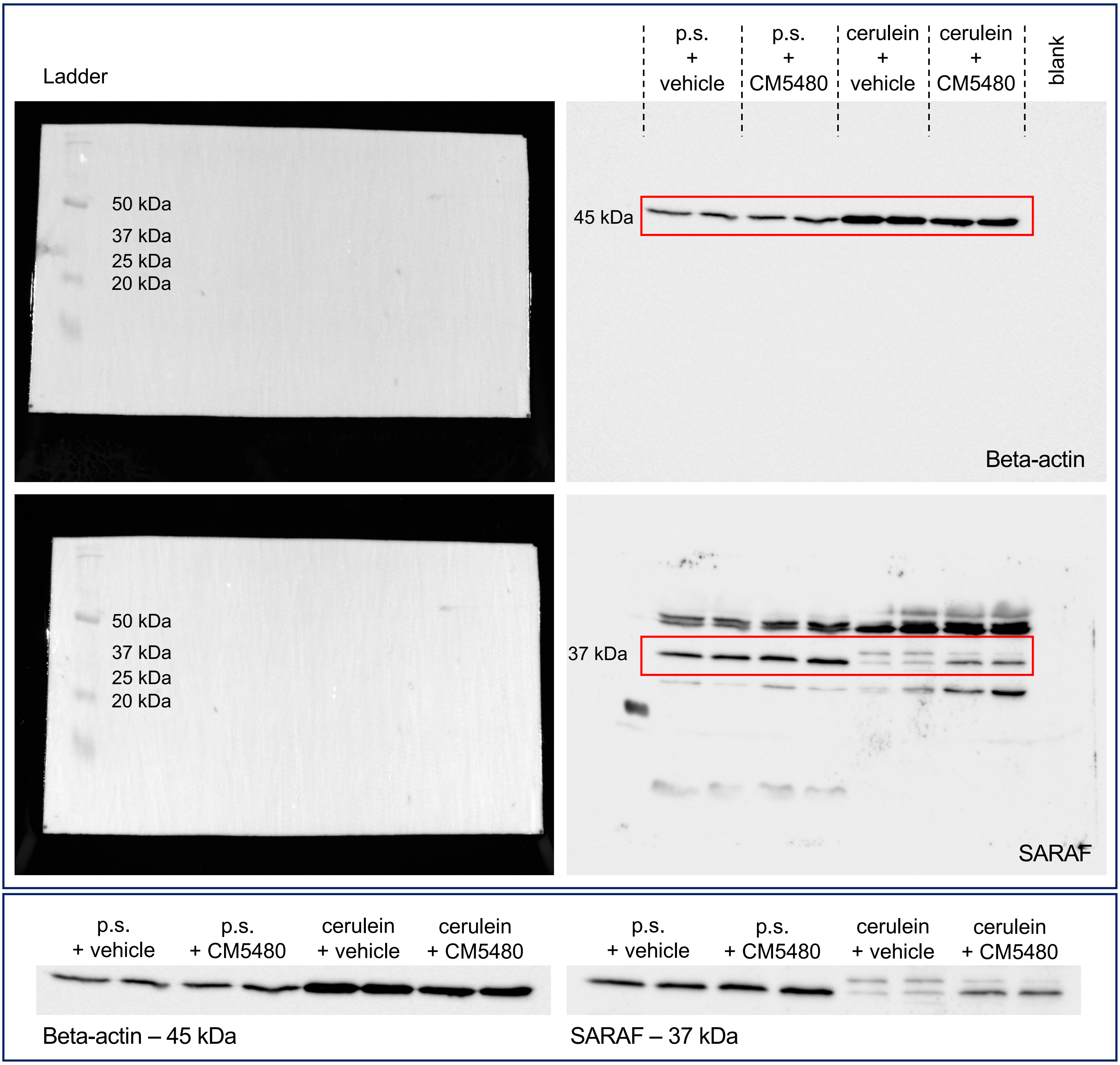

20 µg protein was loaded to the 15% Bis-Tris gel
anti-beta-actin 1:10,000 (overnight) + anti-rabbit HRP secondary 1:10,000 (1hr) – 2 min exposition time
anti-TMEM66 (SARAF) 1:1000 (overnight) + anti-rabbit HRP secondary 1:10,000 (1hr) – 20 sec exposition time

Full unedited blots for Figure 4 showing the western blot analysis of SARAF and beta-actin protein level: protein samples were isolated from mouse pancreas in each group and loaded in 20 µg (*n=6*/group). Red frames indicate which bands were used for the Figure 4.

Antibodies:
anti–TMEM66 (SARAF) (Invitrogen, PA5-31588)
anti-beta-actin (Cell Signaling Technology, 4967S)
anti-rabbit–HRP (Goat Anti-Rabbit HRP conjugate, Invitrogen, 31460)
